# Prevalence of antipsychotic polypharmacy in patients with schizophrenia and other psychotic disorders in the MENAT and EMRO countries: a systematic review and meta-analysis

**DOI:** 10.3389/fpsyt.2026.1792876

**Published:** 2026-04-29

**Authors:** Mohammed A. Alhassan, Mohammed A. Alarabi, Waled M. Albalawi, Thenuwara Arachchige Omila Kasun Meetiyagoda, Anum Nisar, Wondim Ayenew, Fatimah A Shehab, Gary Remington

**Affiliations:** 1 Department of Medical Specialties, College of Medicine, Majmaah University, Al Majmaah, Saudi Arabia; 2 Department of Psychiatry, College of Medicine, King Saud University, Riyadh, Saudi Arabia; 3 King Salman Armed Forces Hospital, Tabuk, Saudi Arabia; 4 Saitama University, Saitama, Japan; 5 Institute of Population Health, University of Liverpool, Liverpool, United Kingdom; 6 Department of Social and Administrative Pharmacy, School of Pharmacy, College of Medicine and Health Sciences, University of Gondar, Gondar, Ethiopia; 7 King Saud Medical City, Riyadh, Saudi Arabia; 8 Schizophrenia Division, Centre for Addiction and Mental Health (CAMH), Toronto, ON, Canada; 9 Department of Psychiatry, University of Toronto, Toronto, ON, Canada; 10 Institute of Medical Sciences, University of Toronto, Toronto, ON, Canada

**Keywords:** antipsychotic polypharmacy, clozapine, EMRO, MENAT, meta-analysis, schizophrenia, systematic review

## Abstract

Antipsychotic monotherapy is considered the standard for schizophrenia treatment. However, many patients with schizophrenia and other psychotic disorders receive antipsychotic polypharmacy (APP). This can be associated with increased adverse effects, drug interactions, and treatment costs. This review aims to synthesize evidence on the prevalence of APP in the Middle East, North Africa, Turkey, and the Eastern Mediterranean (MENAT/EMRO) countries. Eight databases were systematically searched for studies published up to February 2026 that reported APP prevalence among patients with schizophrenia and psychotic disorders in MENAT and EMRO countries. The methodological quality was assessed using the Joanna Briggs Institute (JBI) Critical Appraisal Checklist, while the overall certainty of evidence was evaluated using GRADE conceptual approach (Grading of Recommendations Assessment, Development and Evaluation). The pooled prevalence was estimated using random-effects meta-analysis, and statistical heterogeneity was assessed using tau-squared (τ²) and I² statistic. Potential sources of heterogeneity were explored through subgroup and meta-regression analyses, and publication bias was assessed using funnel plots and Egger’s tests. Seventeen studies with 6,053 individuals with schizophrenia and other psychotic disorders were included. The pooled prevalence of APP was 50% (95% CI: 37%–62%), with substantial heterogeneity (I² = 98.4%), and was commonly associated with second-generation antipsychotics. Evidence linking APP to specific demographic and clinical variables was limited to a few studies, which found APP associated with higher number of hospitalizations. A pooled prevalence across nine of the APP studies revealed that only 12% (95% CI: 8% to 18%) were receiving clozapine. APP is highly prevalent in MENAT/EMRO countries. Given the potential harms of polypharmacy and the limited evidence supporting it, we recommend efforts to reduce APP and increase clozapine utilization among eligible patients. Evidence-based guidelines, clinician training, and improved clozapine accessibility are crucial to optimizing schizophrenia management in the region.

## 1 Introduction

Schizophrenia is a chronic and severe mental disorder characterized by positive symptoms such as hallucinations, delusions, and disorganized thinking, along with negative symptoms including inability to initiate and complete goal-directed activities, reduced speech, and blunted emotional expression (1). According to recent statistics from the World Health Organization (WHO), schizophrenia affects more than 23 million people globally, corresponding to approximately 1 in every 345 individuals (2). It is consistently ranked among the top ten causes of disability worldwide (3), imposes a substantial burden on patients, families, and healthcare systems through significant impairment in social, occupational, educational, and personal functioning (4). The mainstay of schizophrenia management is adequate and appropriate treatment with antipsychotic medication. Possibly due to the considerable proportion of patients who do not achieve adequate symptom control with antipsychotic monotherapy, prescribers sometimes resort to the use of antipsychotic polypharmacy (APP), a practice broadly defined as the concurrent prescription of two or more antipsychotic medications (5).

One of the clinical circumstances leading to APP is symptom resistance, which is encountered in patients with treatment-resistant schizophrenia (TRS). TRS is commonly defined as an inadequate response to at least two antipsychotic trials of sufficient dose and duration, with persistent positive symptoms (6). Nevertheless, across different regions, clinical practice guidelines generally recommend optimized antipsychotic monotherapy and early initiation of clozapine in cases of TRS, rather than antipsychotic combinations. The American Psychiatric Association (APA) Practice Guidelines and the Florida Best Practice Guidelines do not endorse APP, instead recommending symptom-specific adjunctive treatments if there is an inadequate response (7). Similarly, the United Kingdom’s National Institute for Health and Care Excellence (NICE) guidelines discourage combined antipsychotic use, except for short-term cross-titration, and permit clozapine augmentation in cases of TRS only after a comprehensive evaluation of clozapine nonresponse (8). In contrast, the Royal Australian and New Zealand College of Psychiatrists (RANZCP) guidelines allow consideration of APP following the failure of two adequate antipsychotic trials, in cases of ineligibility to clozapine, with close monitoring and regular review for treatment simplification (9). Therefore, antipsychotic monotherapy remains the recommended treatment for most patients with schizophrenia, and clozapine remains the best intervention for patients with TRS (10).

Despite these recommendations, APP continues to be widely practiced across clinical settings worldwide. Ideally, APP should demonstrate superior therapeutic outcomes compared with monotherapy to support its common use, as well as improved outcomes, such as reduced mortality rates and a lower risk of non-psychiatric hospitalizations (11, 12). However, the evidence supporting the efficacy of APP is very limited, showing some benefit to patients with higher symptom severity, and is not without risk of intolerable side effects (13). Indeed, the literature indicates that polypharmacy is frequently associated with adverse health outcomes, particularly among older adults with multiple comorbidities, such as an increased risk of mortality, falls, drug–drug interactions, and medication non-adherence, raising significant concerns about safety and long-term impact on patient well-being (14–16). Numerous studies have examined the prevalence of APP across regions, and the overall literature suggests that it is both widespread and increasing over time. Global analyses, systematic reviews, and large meta-analytic data syntheses have consistently shown that APP remains common across diverse clinical settings and populations, including older adults and inpatients, with region-specific studies from North America, Europe, Africa, and Greenland supporting the same general pattern (11, 16–20). Taken together, these findings indicate that despite guideline recommendations discouraging its routine use, APP has become an increasingly common practice worldwide.

Research on APP remains limited in the Middle East, North Africa, and Turkey (MENAT), and the Eastern Mediterranean Region (EMRO). These regions are characterized by unique demographic, cultural, and healthcare system dynamics that may influence prescribing behaviors and treatment outcomes. For example, factors including variable access to second-generation antipsychotics, limited clozapine monitoring infrastructure, stigma surrounding mental illness, and differences in psychiatric training and policy frameworks may contribute to higher rates of APP and underutilization of clozapine. To date, no comprehensive systematic review or meta-analysis has synthesized the prevalence, prescribing patterns, or determinants of APP within the whole region (17). A systematic understanding of APP in MENAT and EMRO countries is essential to inform regional mental health policies and support the development of targeted interventions to improve treatment outcomes for individuals with schizophrenia (12). Therefore, the primary aim of this systematic review and meta-analysis was to estimate the pooled prevalence of APP among patients with schizophrenia and other psychotic disorders across countries in the MENAT and EMRO regions. The secondary aims were to characterize APP prescribing patterns and assess the utilization of clozapine in the region.

## 2 Methodology

### 2.1 Protocol and registration

This systematic review and meta-analysis followed the Preferred Reporting Items for Systematic Reviews and Meta-Analyses (PRISMA 2020) guidelines (18). The review protocol was registered in the International Prospective Register of Systematic Reviews (PROSPERO) (CRD420251153205).

### 2.2 Eligibility criteria

Studies were included if they met the following criteria:

1. Reported data on APP among adults diagnosed with schizophrenia or schizophrenia spectrum and other psychotic disorders based on standardized diagnostic criteria (DSM or ICD).
2. Conducted in MENAT or EMRO countries.
3. Employed cross-sectional, cohort, or registry-based designs.
4. Reported sufficient data to calculate APP prevalence or determinants.

Studies focusing on disorders other than schizophrenia or schizophrenia spectrum and other psychotic disorders, lacking quantitative data, or published as reviews, or editorials were excluded.

### 2.3 Information sources and search strategy

A comprehensive search was conducted in PubMed, Scopus, Web of Science, Embase, PsycINFO, Medline (via OVID), as well as ClinicalTrials.gov and the regional database QScience. In addition, a grey literature search was performed from Google Scholar. No lower date limit was applied, and the search included all records available up to February 2026. The search strategy, developed in consultation with a medical librarian, combined controlled vocabulary (e.g., MeSH terms) with free-text keywords related to antipsychotic polypharmacy, schizophrenia/schizophrenia spectrum, and MENAT/EMRO countries. Additional articles were identified through citation tracking and screening of reference lists. A detailed description of the database-specific queries and search outcomes is presented in **Supplementary Table 1**.

### 2.4 Study selection and data extraction

Two reviewers (W.A. & T.A.O.K.M.) independently screened all titles, abstracts, and full texts using Rayyan software. The disagreements were resolved through consensus or consultation with a third reviewer (M.A.Alh). A standardized data extraction sheet was used to collect information on study characteristics, participant demographics, the prevalence and characteristics of APP, and the prevalence of clozapine use.

### 2.5 Quality assessment

The Joanna Briggs Institute (JBI) Critical Appraisal Checklist for prevalence studies was used to evaluate the methodological quality of included studies (19). Nine methodological domains were assessed: (1) the appropriateness of the sampling frame to address the target population, (2) whether study participants were sampled in an appropriate manner, (3) the adequacy of the sample size, (4) the clarity in describing study subjects and the study setting, (5) whether the data analysis was conducted with sufficient coverage of the identified sample, (6) the use of valid methods for identifying the condition of interest, (7) whether the condition was measured in a standard and reliable manner for all participants, (8) the appropriateness of the statistical analysis, and (9) whether the response rate was adequate and, if not, whether it was managed appropriately.

Each domain was rated as “Yes,” “No,” or “Unclear.” A rating of “Yes” indicated that the study met the criterion, “No” indicated that the criterion was not met, and “Unclear” indicated insufficient information to determine whether the criterion was satisfied. Based on the number of domains meeting the criteria, studies were categorized into three levels of methodological quality: low risk of bias (7–9 criteria met), moderate risk of bias (4–6 criteria met), and high risk of bias (≤3 criteria met). Discrepancies between reviewers were resolved through discussion.

The certainty of evidence for the main outcomes was further evaluated using the GRADE conceptual approach (Grading of Recommendations Assessment, Development and Evaluation) (20). The certainty of evidence was assessed across several domains, including risk of bias, inconsistency, indirectness, imprecision, and publication bias. Two outcomes were evaluated: the overall prevalence APP and clozapine utilization among individuals with schizophrenia and other psychotic disorders in the MENAT and EMRO regions.

### 2.6 Data synthesis and statistical analysis

All quantitative analyses were performed using R software (version 4.5.1). Meta-analyses and graphical outputs were primarily conducted in R using the meta package.

The primary outcome was the pooled prevalence of APP among patients with schizophrenia in MENAT and EMRO countries. The secondary outcome was the pooled prevalence of clozapine utilization in studies reporting clozapine prescription rates with a clearly defined denominator. The primary pooling approach for both APP and clozapine prevalence was a random-effects model using DerSimonian Laird estimator for tau-squared (τ²), implemented in the meta package in R. Prevalence proportions were analyzed on the logit scale with inverse-variance weighting, and 95% confidence intervals were computed using Hartung-Knapp adjustment under the random-effects model. A random-effects meta-analysis was performed to account for expected between-study heterogeneity. Between-study heterogeneity was estimated using a DerSimonian Laird estimator. Pooled proportions were calculated using a generalized linear mixed model with logit transformation to stabilize variances, and estimates were back-transformed for interpretation. Pooled prevalence estimates were expressed with 95% confidence intervals (CIs). Statistical heterogeneity was assessed using the τ² statistics, where higher values indicate greater between-study variability, and the I² statistic, with values of 25%, 50%, and 75% representing low, moderate, and high heterogeneity, respectively.

To explore potential sources of heterogeneity, we conducted subgroup analyses, meta-regression, and leave-one-out sensitivity analyses. Subgroup analyses were conducted based on country, study design, study setting, diagnosis criteria, and risk of bias. Meta-regression analyses were performed using the meta package under a random-effects model to assess the influence of continuous or categorical covariates, including study year, average age of patients, and proportion of male in each study, on the pooled prevalence estimate. The heterogeneity was estimated using the Restricted Maximum Likelihood method.

Publication bias was assessed using visual inspection of funnel plots and Egger’s regression test. Asymmetry was interpreted as evidence of small-study effects or publication bias. Sensitivity analyses were conducted to assess the accuracy of pooled estimates. A leave-one-out analysis sequentially excluded each study to evaluate its impact on the overall estimate and heterogeneity. Diagnostic Baujat plots were used to identify studies that contributed disproportionately to heterogeneity or to effect size. All statistical tests were two-tailed, with a p-value < 0.05 considered statistically significant (except for heterogeneity tests, where p < 0.10 was used).

## 3 Results

### 3.1 Search results and study characteristics

**Figure 1:**
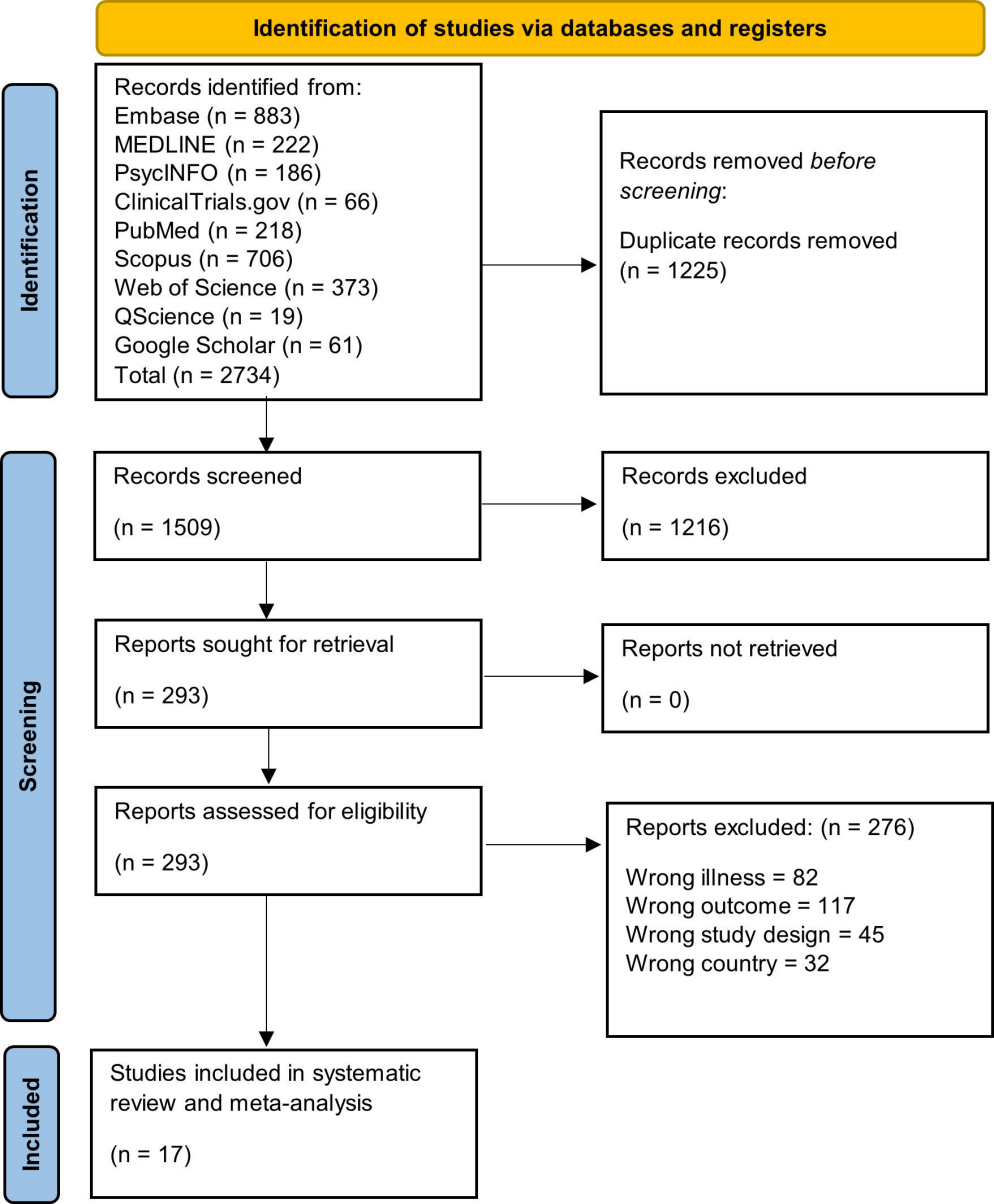
PRISMA flow diagram of study selection for the systematic review and meta-analysis of antipsychotic polypharmacy in individuals with schizophrenia and other psychotic disorders in the MENAT and EMRO regions.

The database search initially identified 2,734 studies, of which 1,509 remained after removal of duplicates. Following title and abstract screening and full-text assessment, 17 studies met the inclusion criteria for this systematic review (**Figure 1**). All 17 studies were eligible for the primary APP meta-analysis, while nine studies were eligible for the secondary clozapine utilization meta-analysis.

The included studies comprised with 6,053 individuals and investigated APP practices among patients with schizophrenia and other psychotic disorders across seven countries in the region of interest, including Saudi Arabia, Turkey, Palestine, Pakistan, Egypt, Qatar, and Jordan. The majority of studies employed a cross-sectional design. The study settings varied, with the majority conducted in outpatient psychiatric clinics, followed by inpatient setting.

**Table 1** summarizes the demographic and clinical characteristics of the included studies. Sample sizes ranged from 42 to 934 participants, which reflects substantial heterogeneity in study scope. Diagnostic criteria were predominantly based on the DSM-IV, DSM-5, or ICD-10 frameworks, although a few studies did not specify their diagnostic reference. The mean age of participants ranged from 32.2 to 41.9 years, indicating that most cohorts consisted of young adults. Males represented the majority of participants across studies, comprising 47–80% of samples. The reported treatment duration ranged from 12 days to 21.2 months (**Table 1**).

**Table 1:** Demographic and Clinical Characteristics of Studies Included in the Systematic Review andMeta-Analysis of Antipsychotic Polypharmacy in Individuals with Schizophrenia and Other Psychotic Disorders in the MENAT and EMRO Regions.

Author (Year)	Country	Study design	Settings	Diagnostic criteria	N	Age	% Male	Treatment duration	APP prevalence	Clozapine prevalence
AlDosari et al. (2025) (59)	Saudi Arabia	Cross-sectional	Outpatient	DSM-5	42	35.7 ± 11.1	50%	21.2 months	Yes	Yes
Alkhadhari et al. (2015) (60)	Egypt	Cross-sectional	Inpatient	DSM-IV	480	32.2 ± 11.4	77%	27.0 ± 21.9 days	Yes	NR
Alkhadhari et al. (2015) (60)	Saudi Arabia	Cross-sectional	Inpatient	DSM-IV	333	36.8 ± 10.9	80%	41.5 ± 42.8 days	Yes	NR
Amr et al. (2012) (21)	Egypt	Cross-sectional	Inpatient	DSM-IV	85	33.2 ± 3.4	61%	34.0 ± 40.0 days	Yes	Yes
Amr et al. (2012) (21)	Jordan	Cross-sectional	Inpatient	DSM-IV	77	33.7 ± 4.1	65%	12.0 ± 15.6 days	Yes	Yes
Atik et al. (2008) (23)	Turkey	Retrospective cross-sectional	Mixed	DSM-IV	122	NR	NR	NR	Yes	NR
Dağdemir et al. (2025) (22)	Turkey	Retrospective cross-sectional	Inpatient	ICD-9/ICD-10	934	34.4 ± 11.2	64%	NR	Yes	Yes
Hattab et al. (2023) (24)	Palestine	Retrospective cross-sectional	Outpatient	Not specified	668	NR	NR	NR	Yes	NR
Kahve et al. (2020) (25)	Turkey	Retrospective cohort	Inpatient	ICD-10	599	35.3 ± 11.7	63%	21 months	Yes	NR
Karadag et al. (2012) (61)	Turkey	Prospective cohort	Mixed	DSM-IV	192	37.6 ± 12.6	54%	NR	Yes	Yes
Khan et al. (2018) (62)	Pakistan	Prospective cohort	Outpatient	ICD-10	72	33.6 ± 11.0	67%	NR	Yes	NR
Khdour et al. (2022) (63)	Palestine	Cross-sectional	Outpatient	Not specified	130	41.8 ± 9.8	60%	NR	Yes	NR
Malik et al. (2019) (64)	Pakistan	Cross-sectional	Mixed	DSM-5/ICD-10	298	NR	56%	NR	Yes	Yes
Ouanes et al. (2020) (12)	Qatar	Retrospective cross-sectional	Mixed	Not specified	306	NR	NR	NR	Yes	NR
Sweileh et al. (2013) (65)	Palestine	Cross-sectional	Inpatient	DSM-IV	250	41.9 ± 11.8	73%	NR	Yes	Yes
Ucok et al. (2007) (66)	Turkey	Cross-sectional	Outpatient	DSM-IV	827	33.1 ± 7.4	66%	NR	Yes	Yes
Yang et al. (2018) (67)	Pakistan	Cross-sectional	Mixed	Not specified	298	36.9 ± 11.9	56%	NR	Yes	Yes
Yazici et al. (2017) (26)	Turkey	Cross-sectional	Outpatient	DSM-5	280	40.9 ± 9.9	66%	81.8 ± 105.7 days	Yes	NR
Yildiz et al. (2003) (68)	Turkey	Retrospective cohort	Outpatient	Not specified	60	34.2 ± 11.8	47%	15.7 ± 10.5 months	Yes	Yes

DSM-5, Diagnostic and Statistical Manual of Mental Disorders, 5^th^ Edition; ICD-10, International Statistical Classification of Diseases and Related Health Problems 10^th^ Revision; DSM-IV-TR, Diagnostic and Statistical Manual of Mental Disorders, Fourth Edition, Text Revision**;** DSM-IV, Diagnostic and Statistical Manual of Mental Disorders, 4^th^ Edition.

### 3.2 Quality of included studies

The methodological quality of the included studies was evaluated using the JBI Critical Appraisal Checklist. The methodological quality of the included studies was generally acceptable (**Table 2**). Among the 17 studies evaluated, 13 were categorized as having a low risk of bias, 3 as moderate risk, and 1 as high risk of bias. Most studies demonstrated appropriate sampling frames and sampling methods, with the majority clearly describing the study subjects and settings. Additionally, nearly all studies used standard and reliable measurement approaches and applied appropriate statistical analyses. Adequate response rates were reported in most studies.

**Table 2:** Quality assessment of included studies using the Joanna Briggs Institute (JBI) critical appraisal checklist.

Author (Year)	Sampling frame appropriate? (Yes/No/Unclear)	Participants sampled in an appropriate way? (Yes/No/Unclear)	Sample size adequate? (Yes/No/Unclear)	Subjects and setting described in detail? (Yes/No/Unclear)	Analyzed with sufficient coverage of the identified sample (Yes/No/Unclear)	Valid methods used to identify condition? (Yes/No/Unclear)	Condition measured in a standard, reliable way for all participants? (Yes/No/Unclear)	Appropriate statistical analysis? (Yes/No/Unclear)	Adequate response rate? (Yes/No/Unclear)	Risk of Bias
AlDosari et al. (2025) (59)	Yes	Yes	No	Yes	Yes	Yes	Yes	Yes	Yes	Low
Alkhadhari et al. (2015) (60)	Yes	Yes	Yes	Yes	Yes	Yes	Yes	Yes	Yes	Low
Amr et al. (2012) (21)	Yes	Yes	No	Yes	Yes	Unclear	Yes	Yes	Yes	Low
Atik et al. (2008) (23)	Yes	Yes	Yes	Yes	Yes	Unclear	Yes	Yes	Yes	Low
Dağdemir et al. (2025) (22)	Yes	Yes	Yes	Yes	Yes	Yes	Yes	Yes	Yes	Low
Hattab et al. (2023) (24)	Yes	Yes	Yes	Unclear	Yes	No	No	Yes	Yes	Moderate
Kahve et al. (2020) (25)	Yes	Yes	Yes	Yes	Yes	Yes	Yes	Yes	Yes	Low
Karadag et al. (2012) (61)	Yes	Yes	No	Yes	Yes	Yes	Yes	Yes	Yes	Low
Khan et al. (2018) (62)	Yes	No	No	Yes	No	Yes	Yes	Yes	Yes	Low
Khdour et al. (2022) (63)	Yes	No	No	Unclear	Yes	Unclear	Yes	Yes	Yes	Moderate
Malik et al. (2019) (64)	Yes	Yes	No	Yes	Yes	Yes	Yes	Yes	Yes	Low
Ouanes et al. (2020) (12)	Yes	Yes	Yes	Unclear	No	Yes	Yes	Yes	Yes	Low
Sweileh et al. (2013) (65)	Yes	Yes	No	Yes	Yes	Yes	Yes	Yes	Yes	Low
Ucok et al. (2007) (66)	Yes	No	Yes	Yes	Unclear	Unclear	Yes	Yes	Yes	Low
Yang et al. (2018) (67)	Yes	Unclear	No	Unclear	Unclear	Unclear	Unclear	Yes	Yes	High
Yazici et al. (2017) (26)	Yes	Yes	No	Yes	Yes	Yes	Yes	Yes	Yes	Low
Yildiz et al. (2003) (68)	Yes	Unclear	No	Yes	Yes	Unclear	Yes	Yes	Yes	Moderate

The overall certainty of evidence was further assessed using the GRADE framework. The certainty was rated as “low” for both the overall APP prevalence and clozapine utilization. The evidence for APP prevalence was downgraded due to substantial heterogeneity across studies and imprecision. For clozapine utilization prevalence, the certainty was similarly downgraded because of inconsistency and imprecision (**Table 3**).

**Table 3:** GRADE assessment of the certainty of evidence for pooled prevalence estimates of antipsychotic polypharmacy and clozapine use in individuals with schizophrenia and other psychotic disorders in the MENAT and EMRO regions.

Outcome	Type	Certainty	Justification
Pooled prevalence of antipsychotic polypharmacy	Prevalence	Low	Downgraded due to substantial heterogeneity and imprecision.
Pooled prevalence of clozapine use	Prevalence	Low	Downgraded due to substantial heterogeneity and imprecision.

GRADE, Grading of Recommendations Assessment, Development and Evaluation.

### 3.3 Prevalence of antipsychotic polypharmacy

A total of 17 studies were included in the quantitative synthesis. Across these studies, 6,053 individuals with schizophrenia and other psychotic disorders were included, of whom 3,041 (50%) were prescribed APP. Using a random-effects meta-analysis, the pooled prevalence of APP in the MENAT and EMRO regions was 0.50 (95% CI: 0.37–0.62), indicating that approximately half of patients with schizophrenia and other psychotic disorders were prescribed more than one antipsychotic in the included studies. However, significant heterogeneity was detected across studies (I² = 98.4%, Tau² = 0.9527, Chi² = 983.16, df = 16, p < 0.0001).

**Figure 2:**
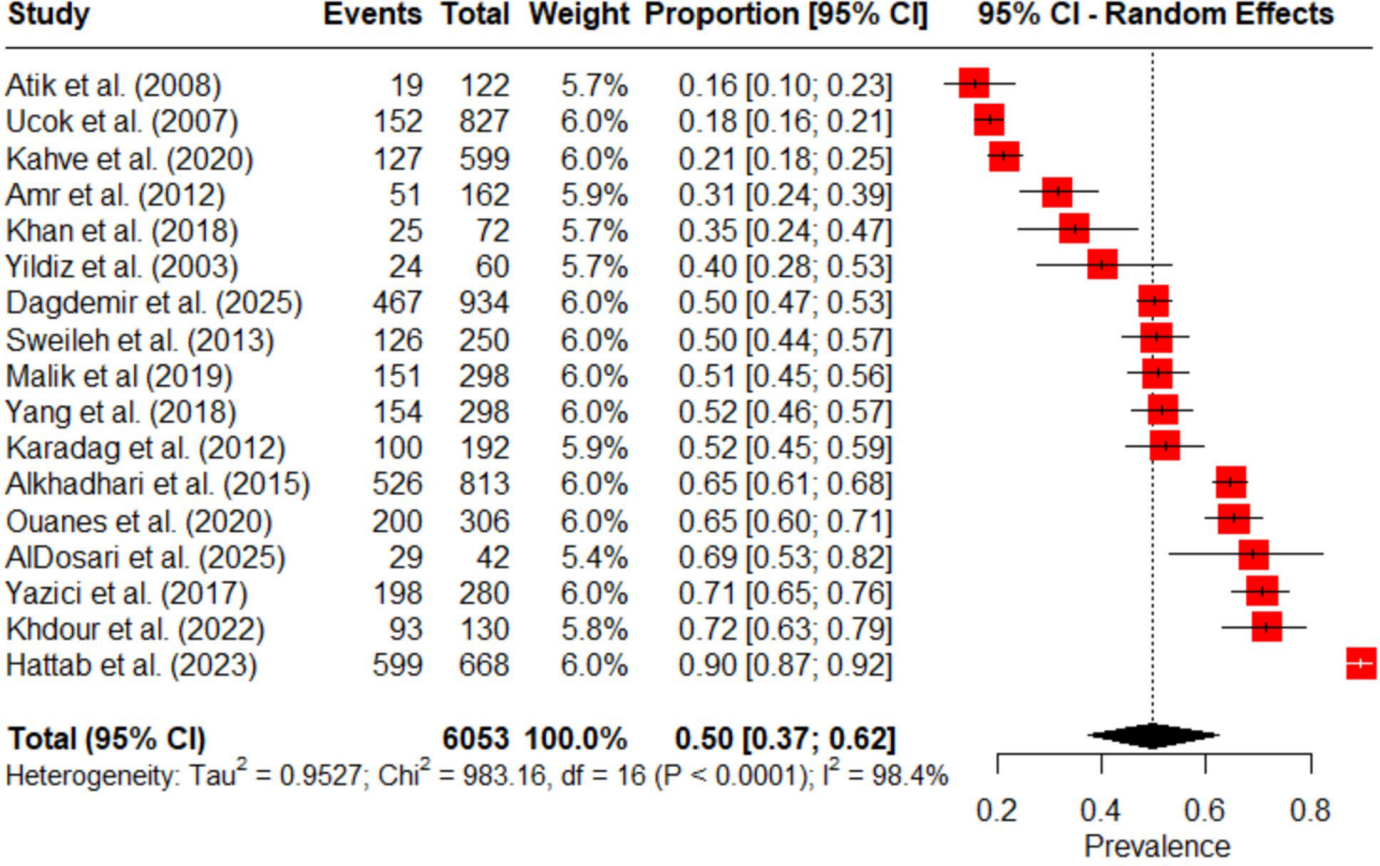
Forest plot of the pooled prevalence of antipsychotic polypharmacy in individuals with schizophrenia and other psychotic disorders in the MENAT and EMRO regions.

The prevalence of APP across studies ranged from 15.6% to 89.6%. The majority of studies defined APP as the concurrent use of two or more antipsychotic medications, although a few studies differed in the required minimum overlap period for co-prescription. **Table 4** summarizes the reported prevalence estimates across studies, while **Figure 2** presents the pooled prevalence of APP with corresponding confidence intervals.

**Table 4:** Studies reporting prevalence of antipsychotic polypharmacy in individuals with schizophrenia and other psychotic disorders in the MENAT and EMRO regions.

Author (Year)	Number of APP events	Prevalence of APP (%)	Definition of APP used
AlDosari et al. (2025) (59)	29	69.0	Patients with schizophrenia administered combination therapy
Alkhadhari et al. (2015) (60) (Egypt)	322	67.1	The use of combinations of antipsychotics (combined therapy)
Alkhadhari et al. (2015) (60) (Saudi Arabia)	204	61.2	The use of combinations of antipsychotics (combined therapy)
Amr et al. (2012) (21) (Egypt)	32	37.6	The concurrent prescription of two antipsychotics
Amr et al. (2012) (21) (Jordan)	19	24.7	The concurrent prescription of two antipsychotics
Atik et al. (2008) (23)	19	15.6	Prescription of more than one antipsychotic prescribed concurrently
Dağdemir et al. (2025) (22)	467	50.0	Concurrent use of two or more antipsychotics
Hattab et al. (2023) (24)	599	89.6	Not clearly mentioned
Kahve et al. (2020) (25)	127	21.2	The use of two antipsychotic drugs at the hospital for two weeks together with an effective dose, or two antipsychotic drugs with an effective dose starting at the hospital while being hospitalized and discharged for ambulatory use
Karadag et al. (2012) (61)	100	52.1	Not mentioned
Khan et al. (2018) (62)	25	34.7	Not mentioned
Khdour et al. (2022) (63)	93	71.5	Not mentioned
Malik et al. (2019) (64)	151	50.6	Prescription of antipsychotics more than one antipsychotic drug
Ouanes et al. (2020) (12)	200	65.3	The use of two or more s antipsychotics
Sweileh et al. (2013) (65)	126	50.4	The use of two or more antipsychotic drugs
Ucok et al. (2007) (66)	152	18.4	Not mentioned
Yang et al. (2018) (67)	154	51.7	The use of two or more antipsychotics
Yazici et al. (2017) (26)	198	70.7	The use of two or more kinds of antipsychotics
Yildiz et al. (2003) (68)	24	40.0	More than one antipsychotic medication prescribed concurrently

### 3.4 Pattern of antipsychotic use in polypharmacy studies

Detailed information on antipsychotic prescribing patterns was reported in nine studies included in the review (**Supplementary Table 2**). Across these studies, considerable variability was found in the classes and combinations of antipsychotics. Second-generation antipsychotics (SGAs) were the most prescribed agents in monotherapy. Several studies reported a predominance of SGA monotherapy, particularly in Saudi Arabia (59), Egypt (60), Pakistan (62), and Palestine (65). Prescribed SGAs included risperidone, aripiprazole, paliperidone, quetiapine, and olanzapine. However, first-generation antipsychotic (FGA) monotherapy remained common in some settings. Studies from Palestine (65) and Pakistan (62) reported substantial use of typical antipsychotics as single-agent therapy.

Regarding polypharmacy, the most frequently reported combinations were SGA + SGA and SGA + FGA. In addition, FGA + FGA combinations were also observed in some cohorts, although less frequently than mixed regimens involving SGAs. A small proportion of patients in some studies were also prescribed three or more antipsychotics concurrently, indicating more complex polypharmacy regimens (65).

### 3.5 Correlates of antipsychotic polypharmacy

Amongst the 17 studies, three studies conducted in four countries included several demographics, clinical, and treatment-related factors examined as potential correlates of APP (**Table 5**). Since the reported variables and models differed substantially across studies, quantitative synthesis was not appropriate.

**Table 5:** Studies reporting correlates of antipsychotic polypharmacy in individuals with schizophrenia and other psychotic disorders in The MENAT and EMRO regions.

Author (Year)	Country	Factor	Value	Reference	OR	Lower	Upper	Significance
Amr et al. (2012) (21)	Egypt, Jordan	Country	Egypt	Jordan	1.14	0.68	1.91	Not significant
		Age	mean years		1.07	0.67	1.7	Not significant
		Education	>= Secondary	< Secondary	0.81	0.52	1.23	Not significant
		Occupation	Working	Not Working	1.04	0.71	1.52	Not significant
		Schizophrenia Type	Paranoid	Not Paranoid	1.15	1.01	1.3	Significant
		Hospitalization	mean number		1.83	1.13	2.95	Significant
		Relapse	mean number		1.89	1.32	2.71	Significant
Dağdemir et al. (2025) (22)	Turkey	Age	mean years		1.01	0.94	1.08	Not significant
		Age of First Psychotic Symptoms	mean years		1	0.93	1.01	Not significant
		Duration of Untreated Psychosis	mean years		1	0.99	1	Not significant
		Hospitalization	mean number		1.01	1.01	1.25	Significant
		Hospitalization	mean days		1.27	0.99	1.62	Not significant
		Longest Remission Period	mean months		1	0.99	1.01	Not significant
		Disease Duration	mean months		1.01	1	1.01	Significant
		Attacks	mean number		1.01	0.9	1.12	Not significant
		Admission Method	Polyclinic	Emergency	0.89	0.64	1.23	Not significant
		Gender	Female	Male	0.68	0.48	0.96	Significant
		Marital Status	Married	Single	0.95	0.67	1.35	Not significant
		Smoker	Yes	No	0.83	0.59	1.17	Not significant
		Medical Comorbidity	Yes	No	1.39	0.85	2.28	Not significant
		Occupation	Working	Not Working	1.47	0.98	2.2	Not significant
		Suicide	Yes	No	0.74	0.48	1.15	Not significant
		Diagnosis Groups	Schizophrenia	Not Schizophrenia	2.05	1.18	3.58	Significant
Sweileh et al. (2013) (65)	Palestine	Gender	Female	Male	1.5	0.88	2.7	Not significant
		Age	mean years		1	0.98	1.03	Not significant
		Marital status	Married	Single	0.8	0.5	1.3	Not significant
		Smoking	Yes	No	1.7	1.02	2.9	Significant
		Occupation	Working	Not Working	1.1	0.5	2.2	Not significant
		Disease Duration	>= 10 years	< 10 years	2	1.2	3.4	Significant
		Hospitalization	>= 2	< 2	2.8	1.5	5.1	Significant
		Family history of DM	Yes	No	0.9	0.42	1.4	Not significant
		Depot use	Yes	No	7.4	4.2	12.9	Significant
		Anticholinergic use	Yes	No	6.7	3.5	12.8	Significant
		SGA use	Yes	No	0.7	0.4	1.2	Not significant

OR, Odds ratio; Value, Category or unit of measurement used for each factor; Reference, Comparator or baseline category against which the reported OR is calculated.

Particularly, clinical illness characteristics appeared to play an important role in APP prescribing patterns. For example, higher numbers of hospitalizations and relapses were associated with increased odds of APP in studies conducted in Egypt and Jordan (21). Similarly, in the Palestinian study, longer disease duration (≥10 years) and having two or more hospitalizations were significantly associated with APP use (65). Treatment-related factors also demonstrated strong associations. The use of depot antipsychotics and anticholinergic medications showed markedly higher odds of APP in the Palestinian cohort. In the Turkish study, a diagnosis of schizophrenia (compared with non-schizophrenia) was significantly associated with APP (22).

### 3.6 Publication bias

**Figure 3:**
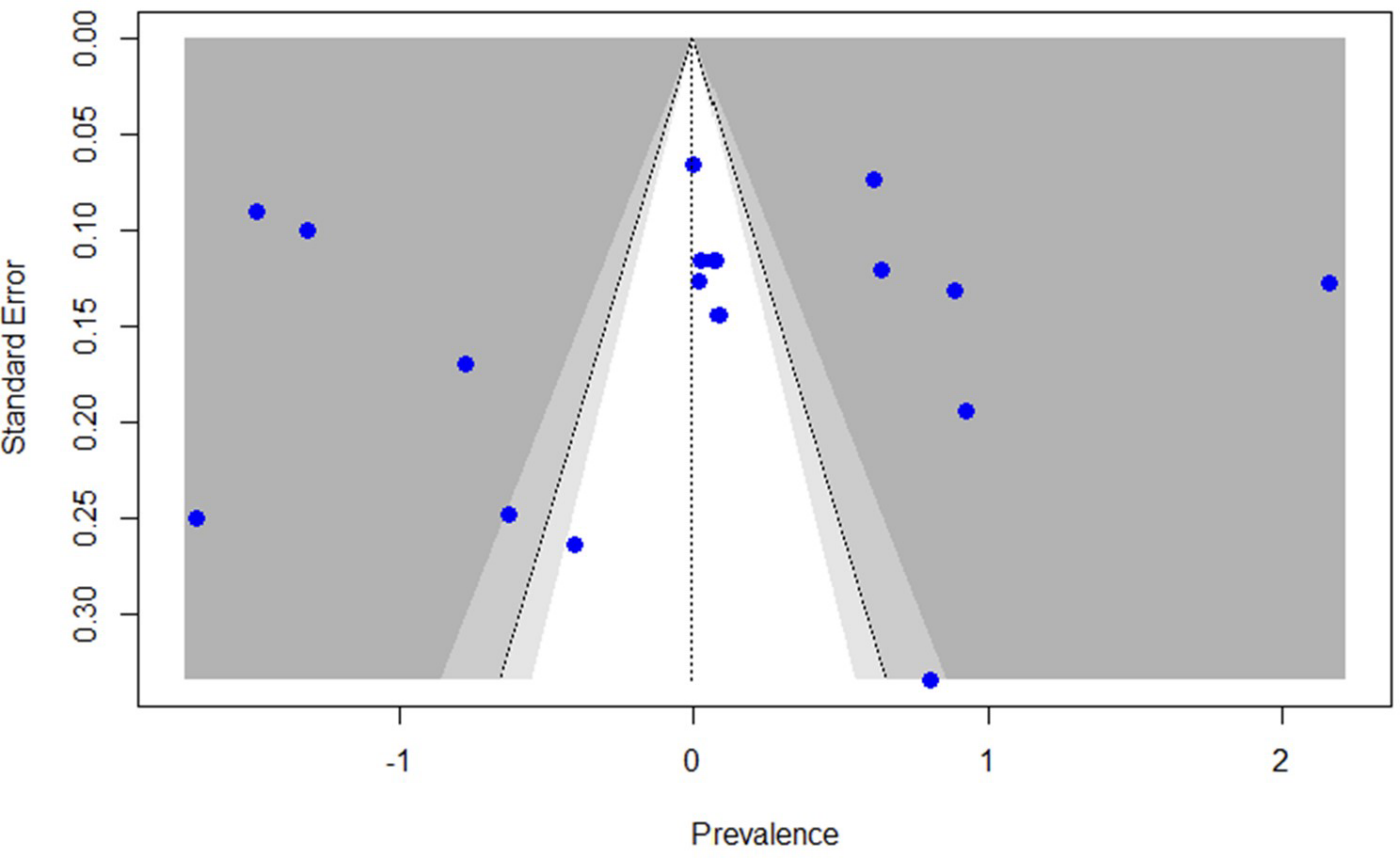
Funnel plot of studies reporting the prevalence of antipsychotic polypharmacy in individuals with schizophrenia and other psychotic disorders in the MENAT and EMRO regions.

In this review and meta-analysis, visual inspection of the funnel plot showed an atypical distribution, with several studies lying outside the 95% confidence limits (**Figure 3**). Given the extreme heterogeneity across studies (I² = 98.4%), both visual inspection of the funnel plot and Egger’s test have limited interpretability in this context. Egger’s test did not indicate statistically significant asymmetry (p = 0.9449). However, in the presence of such high heterogeneity, asymmetry tests may be unreliable and should not be considered definitive evidence for or against publication bias.

### 3.7 Subgroup analysis

A random-effects meta-analysis of the 17 included studies was conducted to examine the pooled prevalence of APP stratified by country, study design, study setting, diagnostic criteria, and risk of bias. Subgroup analyses revealed marked geographic variation: Turkey (7 studies) showed a lower prevalence of 36%, while Palestine (3 studies) had the highest at 74%, although the confidence interval was wide, reflecting limited evidence. Pakistan (3 studies) reported 47% with moderate heterogeneity, and Saudi Arabia (2 studies) yielded a more consistent estimate of 62% with no heterogeneity. Egypt (2 studies, 53%) and the “Others” group comprising Qatar and Jordan (2 studies, 44%) both had wide confidence intervals and high heterogeneity, reflecting limited and variable evidence (**Table 6**). Subgroup analysis by study design indicated that cross-sectional studies reported a higher pooled prevalence (53%) compared to cohort studies (36%) (**Table 7**). However, the small number of cohort studies limits the robustness of the subgroup comparison by study design, and substantial heterogeneity persisted in both groups. In terms of study setting, outpatient studies showed a higher prevalence (58%) than inpatient studies (44%) (**Table 8**). However, heterogeneity remained high and these differences should be interpreted cautiously. Subgroup analysis based on diagnostic criteria showed variability in the prevalence of APP, with the highest prevalence observed in studies using DSM-5 criteria (70%) and the lowest in those using ICD-10 criteria (27%) (**Table 9**), while substantial heterogeneity remained in most subgroups. Similarly, risk of bias subgroup analysis indicated APP prevalence of 45% among low-risk studies and 71% among moderate-risk studies, with consistently high heterogeneity across analyses (**Table 10**). The findings should be interpreted cautiously due to the small number of studies.

**Table 6:** Prevalence of antipsychotic polypharmacy in schizophrenia and other psychotic disorders in the MENAT and EMRO Regions: Country based subgroup analysis.

Country	No. of studies	Prevalence [95% CI]	*τ* ^2^	*I* ^2^	*χ*^2^ p-value
Overall	17	0.50 [0.37, 0.62]	0.95	98.4%	<0.0001
Turkey	7	0.36 [0.19, 0.58]	0.90	98.5%	<0.0001
Palestine	3	0.74 [0.16, 0.98]	1.15	98.6%	<0.0001
Pakistan	3	0.47 [0.26, 0.68]	0.09	70.7%	0.0329
Saudi Arabia	2	0.62 [0.30, 0.86]	0.00	0.0%	0.3287
Egypt	2	0.53 [0.00, 1.00]	0.71	96.0%	<0.0001
Others	2	0.44 [0.00, 1.00]	1.49	97.2%	<0.0001

Studies (60) and (21) included data from two countries. Others combined Qatar and Jordan.

**Table 7:** Prevalence of antipsychotic polypharmacy in schizophrenia and other psychotic disorders in the MENAT and EMRO regions: study design subgroup analysis.

Study design	No. of studies	Prevalence [95% CI]	*τ* ^2^	*I* ^2^	*χ*^2^ p-value
Overall	17	0.50 [0.37, 0.62]	0.95	98.4%	<0.0001
Cross-sectional	13	0.53 [0.39, 0.67]	0.99	98.2%	<0.0001
Cohort	4	0.36 [0.18, 0.59]	0.34	95.5%	<0.0001

**Table 8:** Prevalence of antipsychotic polypharmacy in schizophrenia and other psychotic disorders in the MENAT and EMRO regions: study setting subgroup analysis.

Study settings	No. of studies	Prevalence [95% CI]	*τ* ^2^	*I* ^2^	*χ*^2^ p-value
Overall	17	0.50 [0.37, 0.62]	0.95	98.4%	<0.0001
Inpatient	5	0.44 [0.28, 0.62]	0.56	97.7%	<0.0001
Outpatient	7	0.58 [0.31, 0.81]	1.50	99.1%	<0.0001
Mixed	5	0.46 [0.23, 0.72]	0.70	94.4%	<0.0001

**Table 9:** Prevalence of antipsychotic polypharmacy in schizophrenia and other psychotic disorders in the MENAT and EMRO regions: diagnostic criteria subgroup analysis.

Diagnostic criteria	No. of studies	Prevalence [95% CI]	*τ* ^2^	*I* ^2^	*χ*^2^ p-value
Overall	17	0.50 [0.37, 0.62]	0.95	98.4%	<0.0001
DSM-IV	6	0.39 [0.23, 0.58]	0.79	98.2%	<0.0001
DSM-5	2	0.70 [0.63, 0.77]	0.00	0.0%	0.8252
ICD-10	2	0.27 [0.00, 0.96]	0.20	84.7%	0.0107
Mixed	2	0.50 [0.47, 0.54]	0.00	0.0%	0.8401
Not specified	5	0.67 [0.37, 0.87]	0.92	97.7%	<0.0001

**Table 10:** Prevalence of antipsychotic polypharmacy in schizophrenia and other psychotic disorders in the MENAT and EMRO regions: risk of bias subgroup analysis.

Risk of Bias	No. of studies	Prevalence [95% CI]	*τ* ^2^	*I* ^2^	*χ*^2^ p-value
Overall	17	0.50 [0.37, 0.62]	0.95	98.4%	<0.0001
Low	13	0.45 [0.34, 0.57]	0.71	97.8%	<0.0001
Moderate	3	0.71 [0.09, 0.98]	1.60	97.7%	<0.0001
High	1	0.52 [0.46, 0.57]	–	–	–

### 3.8 Meta regression

To explore potential sources of heterogeneity in APP prevalence, meta-regression analyses were conducted (**Table 11**). Meta-regression indicated that publication year was significantly associated with increasing APP prevalence (p = 0.0142), suggesting more frequent APP in more recent studies.

**Table 11:** Summary results of meta-regression analysis on prevalence of antipsychotic polypharmacy in schizophrenia and other psychotic disorders in the MENAT and EMRO regions.

Variable	k	Estimate	SE	95% CI	p-value	Pre *τ*^2^	Post *τ*^2^	R^2^
Year of Publication (numeric)	17	0.09	0.03	[0.02, 0.16]	0.0142	0.95	0.67	29.8%
Mean Age (numeric)	14	0.01	0.02	[-0.03, 0.05]	0.5371	0.95	0.59	0.0%
% Male (percentage)	14	-0.23	2.59	[-5.87, 5.41]	0.9319	0.95	0.61	0.0%

### 3.9 Sensitivity analysis

**Figure 4:**
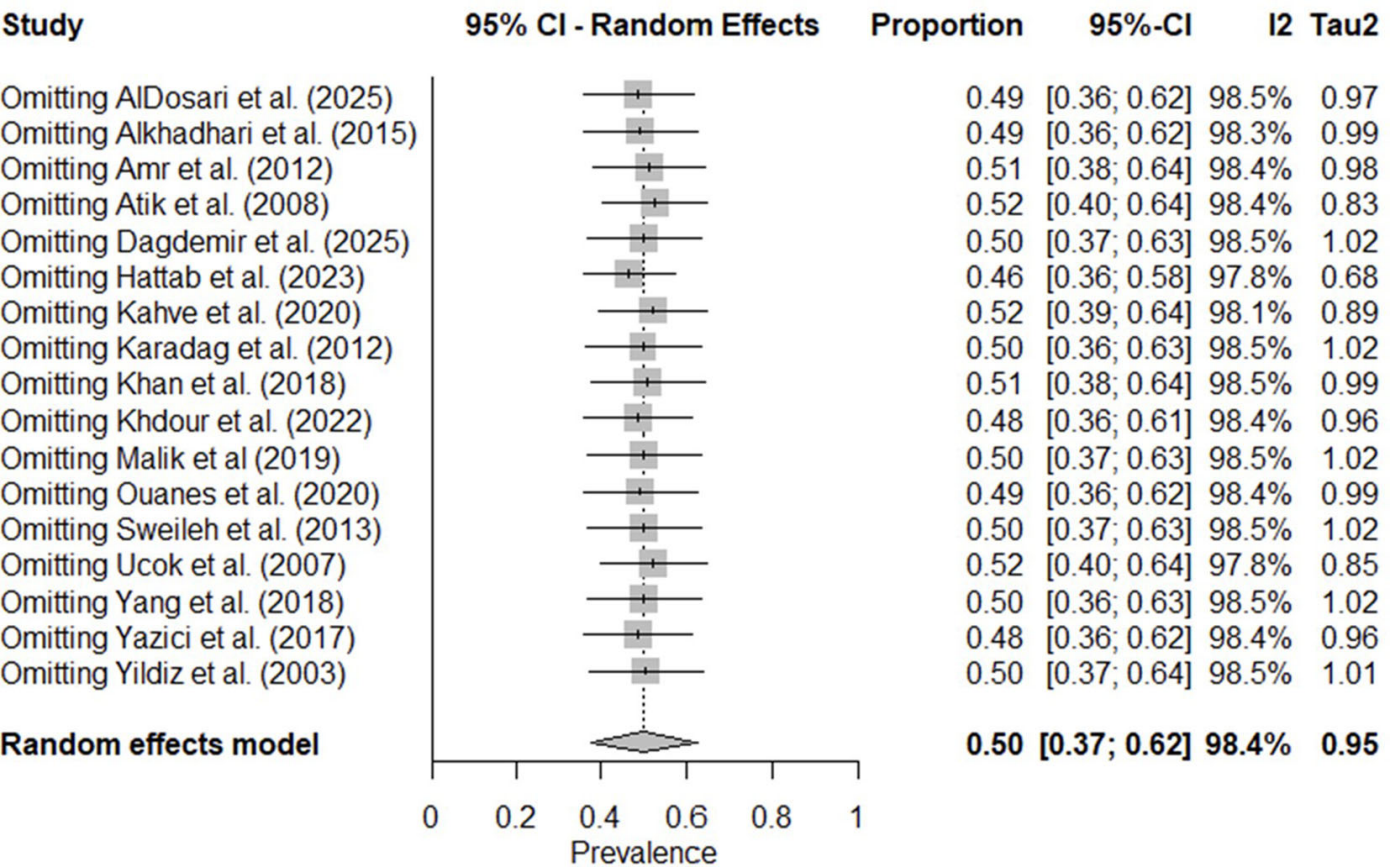
Leave-one-out sensitivity analysis of the pooled prevalence of antipsychotic polypharmacy in Individuals with schizophrenia and other psychotic disorders in the MENAT and EMRO regions.

**Figure 5:**
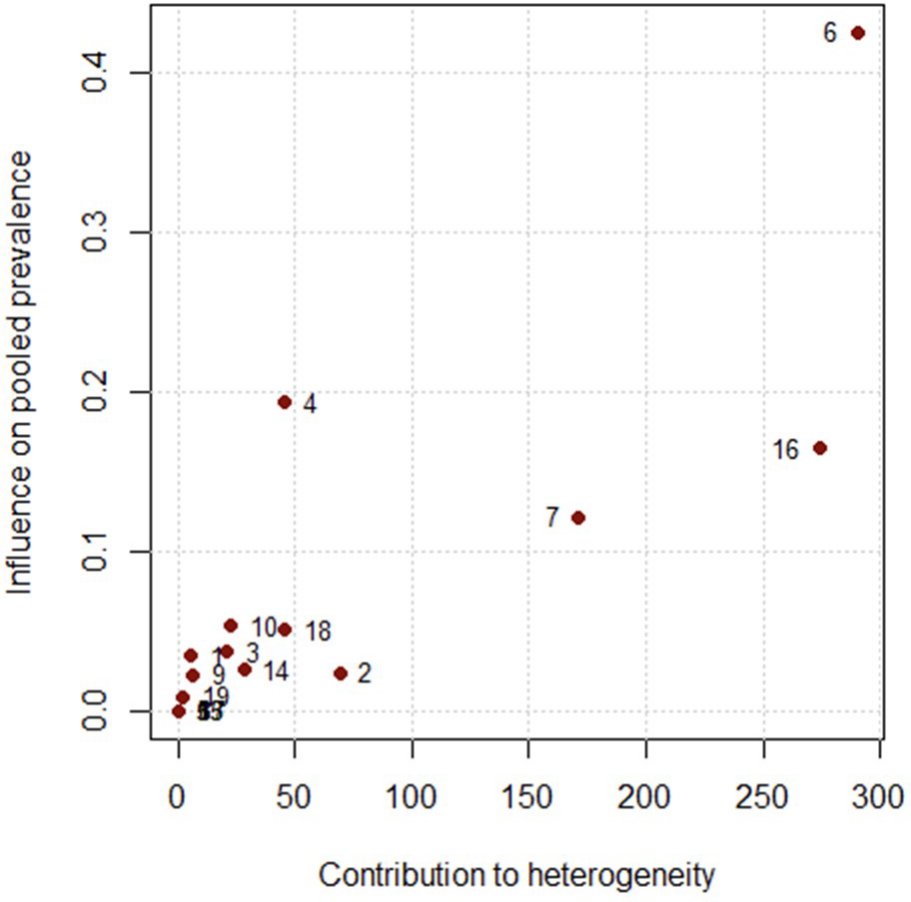
Baujat plot of studies contributing to heterogeneity in the meta-analysis of antipsychotic polypharmacy in individuals with schizophrenia and other psychotic disorders in the MENAT and EMRO regions.

The leave-one-out analysis (**Figure 4**) showed that no single study substantially influenced the pooled prevalence estimate, as I² values remained above 97.8% in all iterations. The diagnostic Baujat plot (**Figure 5**) indicated that multiple studies contributed to the observed heterogeneity, with four studies (23–26), appearing visually distinct from others. These findings suggest that heterogeneity is not driven by a single outlier study but is likely attributable to variations across multiple studies.

### 3.10 Prevalence of clozapine utilization

**Figure 6:**
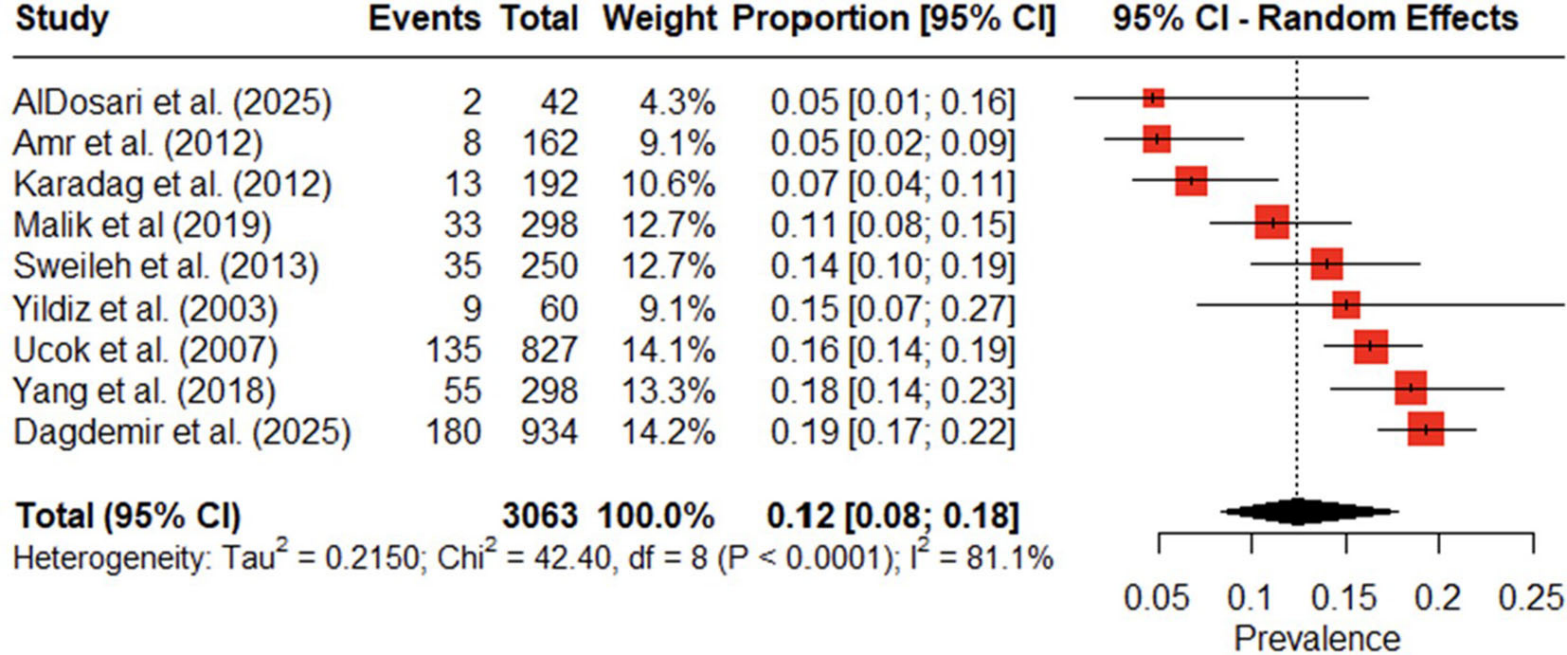
Forest plot of clozapine utilization in antipsychotic polypharmacy studies in individuals with schizophrenia and other psychotic disorders in the MENAT and EMRO regions.

Given the high prevalence of APP observed, we further examined the prevalence of clozapine utilization across included studies. In this review and meta-analysis, nine studies reported clozapine use. The pooled prevalence of clozapine use in MENAT and EMRO regions was 12% (95% CI: 8% to 18%). **Table 12** summarizes the reported clozapine utilization across studies. The forest plot also presents variability in clozapine utilization across countries, illustrating the lack of uniformity in clozapine prescribing across the region (**Figure 6**).

**Table 12:** Studies reporting clozapine utilization in antipsychotic polypharmacy studies in individuals with schizophrenia in the MENAT and EMRO regions.

Author (Year)	Country	Event size	Number of events of clozapine	Prevalence of clozapine utilization (%)
AlDosari et al. (2025) (65)	Saudi Arabia	42	2	4.8
Amr et al. (2012) (21)	Egypt	162	8	4.9
Dağdemir et al. (2025) (22)	Turkey	934	180	19.3
Karadag et al. (2012) (61)	Turkey	192	13	6.8
Malik et al. (2019) (64)	Pakistan	298	33	11.1
Swelieh et al. (2013) (63)	Palestine	250	35	14.0
Yang et al. (2018) (67)	Pakistan	298	55	18.5
Yildiz et al. (2003) (68)	Turkey	60	9	15.0
U cok et al. (2007) (66)	Turkey	827	135	16.3

Overall heterogeneity was extremely high (I² = 81.1%), indicating significant variation in clozapine prevalence across studies. To assess the source of heterogeneity, we conducted a subgroup analysis, meta-regression, and sensitivity analysis. The subgroup analysis showed apparent differences in clozapine utilization across countries. Pakistan and Turkey had slightly higher pooled prevalence (14%), while studies from “other” category included Saudi Arabia, Palestine, Jordan, and Egypt reported a low rate (7%) (**Table 13**).

**Table 13:** Clozapine utilization in schizophrenia and other psychotic disorders in the MENAT and EMRO regions: country subgroup analysis.

Country	No. of studies	Prevalence [95% CI]	*τ* ^2^	*I* ^2^	*χ*^2^ p-value
Overall	9	0.12 [0.08, 0.18]	0.22	81.1%	<0.0001
Turkey	4	0.14 [0.07, 0.27]	0.21	82.2%	0.0008
Pakistan	2	0.14 [0.00, 0.88]	0.15	84.2%	0.0119
Others	4	0.07 [0.03, 0.19]	0.34	68.8%	0.0222

‘Others’ include Saudi Arabia, Palestine, Jordan, and Egypt with one study each.

Subgroup analysis by study design showed that cross-sectional studies reported a clozapine utilization of 13%, while cohort studies showed a slightly lower estimate of 10%, although the number of cohort studies was limited, estimates should be interpreted with caution (**Table 14**). Subgroup analysis by treatment settings demonstrated comparable clozapine utilization across inpatient (10%), outpatient (10%), and mixed settings (12%), with considerable heterogeneity across groups (**Table 15**). Risk of bias subgroup analysis also showed varying prevalence estimates, with clozapine use of 11% in studies with low risk of bias and 15% in studies with moderate risk of bias (**Table 16**).

**Table 14:** Clozapine utilization in schizophrenia and other psychotic disorders in the MENAT and EMRO regions: study design subgroup analysis.

Study design	No. of studies	Prevalence [95% CI]	*τ* ^2^	*I* ^2^	*χ*^2^ p-value
Overall	9	0.12 [0.08, 0.18]	0.22	81.1%	<0.0001
Cross-sectional	7	0.13 [0.08, 0.19]	0.20	77.0%	<0.0001
Cohort	2	0.10 [0.00, 0.97]	0.29	72.9%	0.0545

**Table 15:** Clozapine utilization in schizophrenia and other psychotic disorders in the MENAT and EMRO regions: study setting subgroup analysis.

Study setting	No. of studies	Prevalence [95% CI]	*τ* ^2^	*I* ^2^	*χ*^2^ p-value
Overall	9	0.12 [0.08, 0.18]	0.22	81.1%	<0.0001
Inpatient	3	0.10 [0.03, 0.29]	0.50	84.3%	0.0003
Outpatient	5	0.10 [0.05, 0.19]	0.25	96.9%	<0.0001
Mixed	3	0.12 [0.03, 0.35]	0.27	86.5%	0.0006

**Table 16:** Clozapine utilization in schizophrenia and other psychotic disorders in the MENAT and EMRO regions: risk of bias subgroup analysis.

Risk of bias	No. of studies	Prevalence [95% CI]	*τ* ^2^	*I* ^2^	*χ*^2^ p-value
Overall	9	0.12 [0.08, 0.18]	0.22	81.1%	<0.0001
Low	7	0.11 [0.07, 0.17]	0.27	82.9%	<0.0001
Moderate	1	0.15 [0.08, 0.26]	–	–	–
High	1	0.18 [0.14, 0.23]	–	–	–

**Figure 7:**
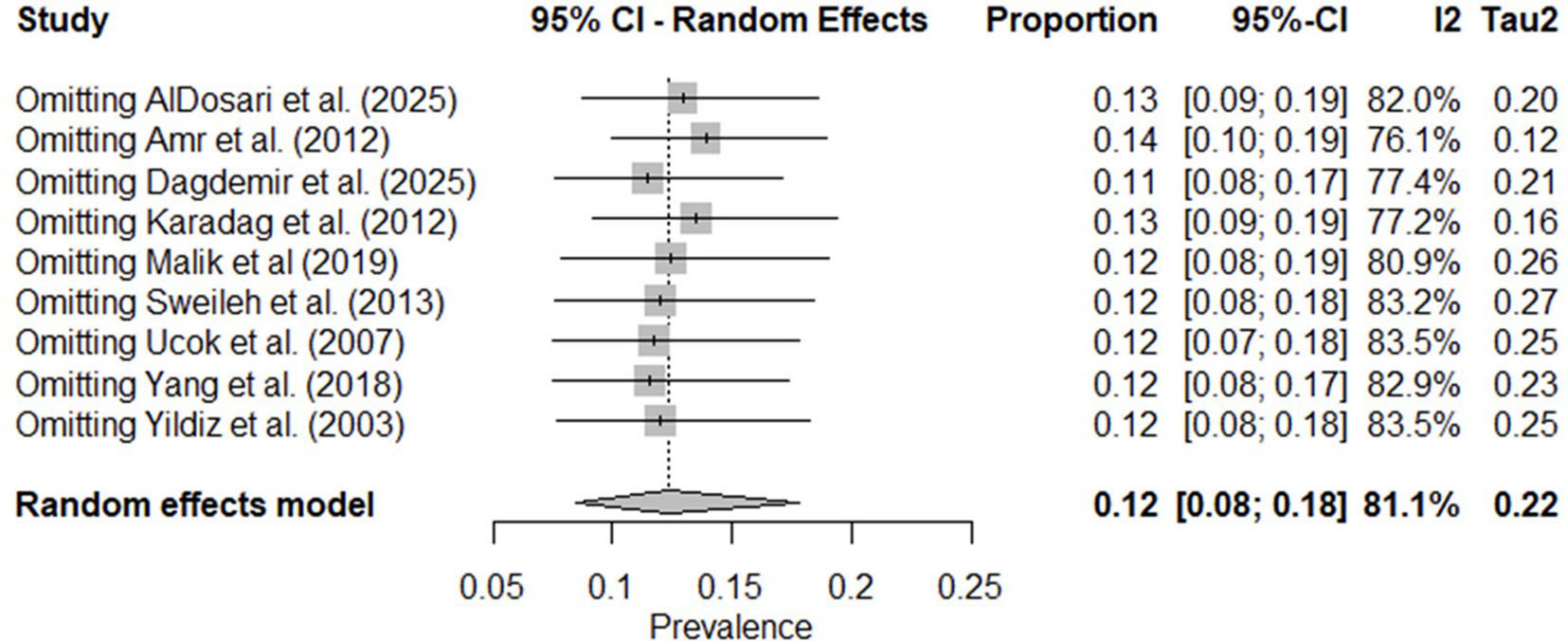
Leave-one-out sensitivity analysis of clozapine utilization in antipsychotic polypharmacy studies in individuals with schizophrenia and other psychotic disorders in the MENAT and EMRO regions.

**Figure 8:**
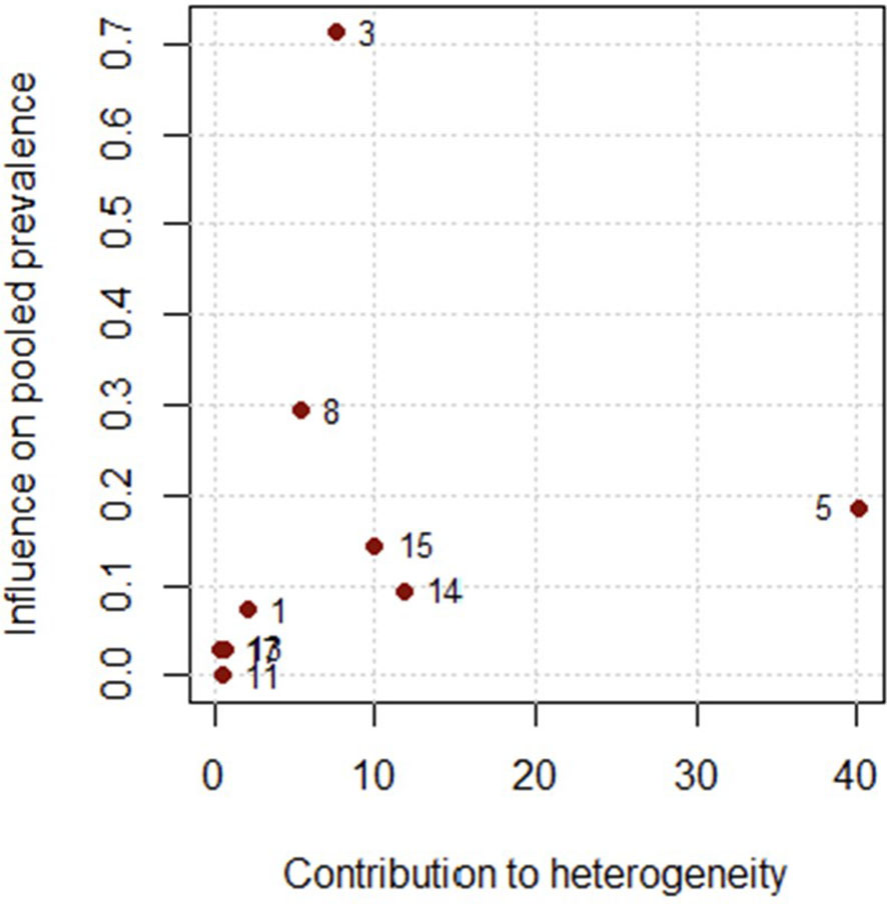
Baujat plot of studies contributing to heterogeneity in the meta-analysis of clozapine utilization in antipsychotic polypharmacy studies in individuals with schizophrenia and other psychotic disorders in the MENAT and EMRO regions.

**Figure 9:**
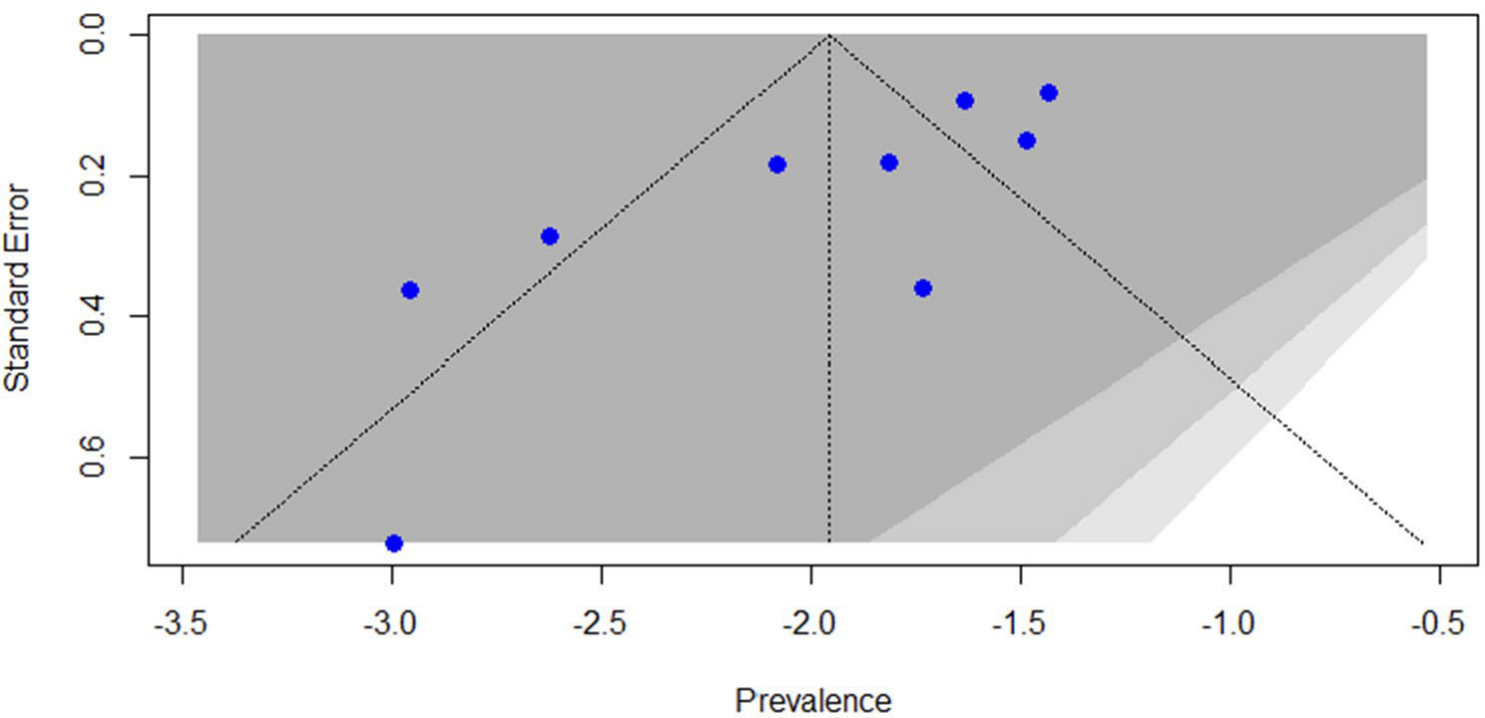
Funnel plot of studies reporting clozapine utilization in antipsychotic polypharmacy studies in individuals with schizophrenia and other psychotic disorders in the MENAT and EMRO regions.

The leave-one-out sensitivity analysis demonstrated that the pooled prevalence of clozapine utilization remained stable regardless of which individual study was removed. All re-estimated pooled effects fell within the range 11.0%–14.0%, and none of the omitted studies caused a substantial shift in either the effect size or the width of the confidence intervals (**Figure 7**). The diagnostic Baujat plot indicated that multiple studies contributed to the observed heterogeneity (**Figure 8**). Furthermore, the meta-regression indicated that study variables such as publication year, mean patient age, and the proportion of male participants were not significantly associated with the prevalence of clozapine use (**Table 17**). Visual inspection of the funnel plot showed an atypical, approximately linear distribution of studies rather than a classical funnel shape (**Figure 9**). Egger’s test indicated statistically significant asymmetry (p = 0.0088). However, given the small number of included studies (k = 9) and the presence of between-study heterogeneity, the reliability of asymmetry tests is limited. The observed pattern is more consistent with small-study effects or methodological differences between studies rather than clear evidence of publication bias.

**Table 17:** Summary results of meta-regression analysis on prevalence of clozapine utilization in schizophrenia and other psychotic disorders in the MENAT and EMRO regions.

Variable	k	Estimate	SE	95% CI	p-value	Pre *τ*^2^	Post *τ*^2^	R^2^
Year (numeric)	9	0.01	0.03	[-0.07, 0.07]	0.8800	0.22	0.26	0.0%
Mean Age (numeric)	9	-0.04	0.02	[-0.08, 0.01]	0.0590	0.22	0.09	56.1%
% Male (percentage)	9	1.75	2.54	[-4.26, 7.76]	0.5124	0.22	0.24	0.0%

## 4 Discussion

The findings of this systematic review suggest that APP in schizophrenia patients is widely practiced across MENAT countries, despite international guidelines consistently recommending antipsychotic monotherapy as the standard of care. Across the included studies, APP prevalence varied substantially, reflecting marked heterogeneity. Such high heterogeneity is common in prevalence meta-analyses and may reflect differences in healthcare systems, prescribing practices, study populations, and clinical settings across countries.

Our findings suggest that APP in the MENAT-EMRO region is higher than that reported in many other parts of the world. Although international estimates vary substantially across countries and regions (27), most published rates from Europe, North America, and parts of Asia tend to fall below the prevalence observed in our analysis (11, 17, 27–29), whereas reports from other countries in Asia and Africa, and estimates from Australia, appear more comparable (30–34). Overall, the global literature points not only to marked geographic variation, but also to a broader pattern of increasing polypharmacy in psychiatric practice. This is consistent with our own results, in which meta-regression showed that year of publication was associated with increasing prevalence of APP. The high prevalence identified in MENAT and EMRO countries places the region toward the upper margin of global estimates, pointing to the possibility that APP is well-established in local psychiatric practice (35). It is plausible to assume that within the region, psychiatrists are accustomed to resort to APP as a strategy to enhance symptom control, manage treatment-resistant cases, or address persistent functional impairments (12, 36).

We found no consistent evidence linking certain demographic, clinical, and treatment-related factors to APP. However, amongst clinical factors, higher numbers of hospitalizations was associated with APP in the three reviewed studies. Along with the frequent use of depot antipsychotics and anticholinergic medications with APP, the findings suggest that APP is more commonly applied in cases perceived as complex, difficult to stabilize, or resistant to standard monotherapy.

We encountered significant heterogeneity across studies, which indicates substantial variability across different study characteristics. The higher prevalence reported in certain countries, such as Palestine and Saudi Arabia, may indicate contextual influences, including prescriber preference (24, 35), diagnostic uncertainty, and financial constraints (37), or differences in medication availability. These findings highlight the need for standardized data reporting and prospective multicenter studies across the region to clarify temporal and clinical determinants of APP use. Variations were also evident by study design, with higher prevalence in cross-sectional studies compared to cohort studies. This reflects methodological differences, as cross-sectional studies and chart reviews are more likely to capture short-term prescribing overlaps or polypharmacy resulting from switching antipsychotics than longitudinal designs. Differences were further observed based on diagnostic criteria, with DSM-5–based studies reporting higher prevalence than those using ICD-10. Furthermore, studies with moderate risk of bias reported higher prevalence compared to low-risk studies. Despite these patterns, substantial heterogeneity persisted across most subgroups, and the findings should be interpreted with caution given the limited number of studies in several categories. Meta-regression revealed publication year was significantly associated with increasing APP prevalence, which indicates a rising trend of APP use in more recent studies. The funnel plot interpretation was inconclusive because of the extreme heterogeneity across studies, although Egger’s test did not detect statistically significant asymmetry. Sensitivity analysis further demonstrated that no single study disproportionately influenced our APP pooled estimate.

**Figure 10:**
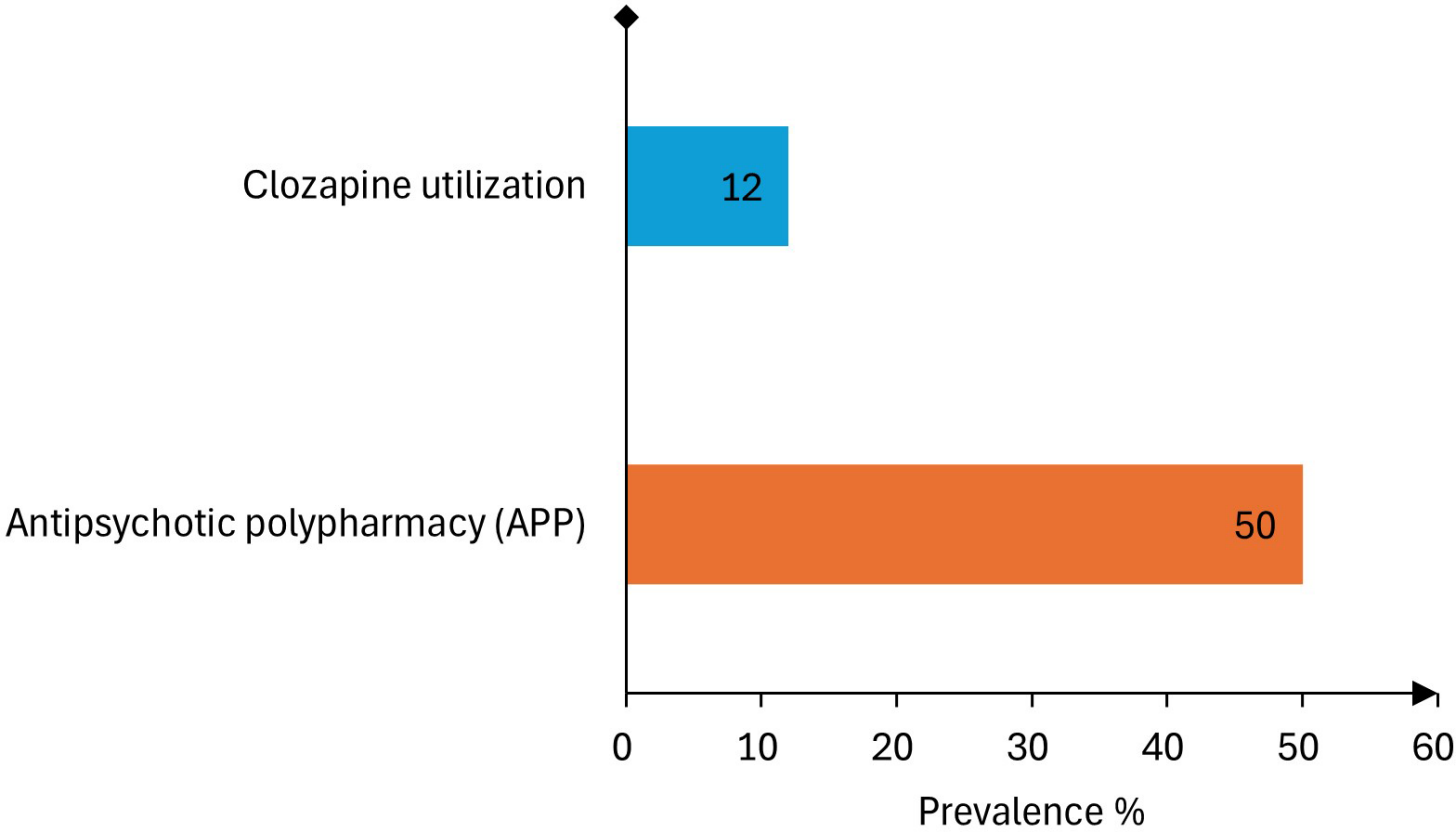
Comparison of antipsychotic polypharmacy and clozapine utilization prevalence among studies reporting antipsychotic polypharmacy in individuals with schizophrenia and other psychotic disorders in the MENAT and EMRO regions.

In parallel with the high APP prevalence of 50%, the pooled prevalence of clozapine utilization within APP studies was only 12% (**Figure 10**), with high heterogeneity, indicating variation across countries and study designs. Considering that TRS affects approximately 22–40% of patients with schizophrenia (38), this prevalence of clozapine use suggests that the medication remains underutilized relative to the expected treatment need. Such quantitative imbalance suggests that APP may be frequently utilized as a substitute for evidence-based clozapine treatment rather than as a last-resort strategy following clozapine failure or intolerability.

Consistent with the low utilization of clozapine in our secondary analysis, a qualitative interview-based study conducted by Ismail et al. reported that there is tendency to under-prescribe clozapine in the Arabian Gulf region (39). Clozapine has been consistently underutilized globally (40), with relatively infrequent utilization in schizophrenia patients living in the United Kingdom (12.8%) (41), Denmark (9.0% to 10.1%) (42), the U.S. (0.9% to 7.8%) (43), Japan (44), and Québec, Canada (3.9% to 9.0%) (45). However, a recent multinational analysis across 75 countries reported that global clozapine utilization increased between 2014 and 2024, corresponding to an approximate 39.3% relative increase worldwide (46). However, in this cited longitudinal study by Fitzgerald et al., Saudi Arabia, Pakistan, and Jordan showed no statistically significant change for clozapine utilization during 2014–2024, whereas significant increases were reported in Egypt, Tunisia, Lebanon, and Turkey (46). This may be due to APP being employed in place of clozapine, despite limited evidence supporting this practice (47).

Given the well-established efficacy of clozapine in TRS, the persistent reliance on APP raises an important question as to why psychiatrists continue to favor antipsychotic combinations over clozapine. This pattern may be partly explained by the multiple barriers that constrain clozapine use, including clinician-related hesitancy, system-level limitations, and patient-related factors (48). For instance, psychiatrists in Iran (49) and Pakistan (50) reported that their underutilization or delayed initiation of clozapine is related to apprehensions about adverse effects, stringent blood monitoring requirements, and limited access to specialized clozapine clinics. In some cases, clinicians favor APP over clozapine due to their fear of hematological and cardiovascular side-effects (51), lack of sufficient training, knowledge and confidence (52) of prescribing clozapine, or paternalistic concern regarding patient attitudes (53). Importantly, delays in clozapine initiation are often associated with limited adherence to national and international clinical guidelines by clinicians (47). Moreover, inadequate mental health infrastructure and uneven access (54) to psychiatric services further hinder optimal treatment delivery and contribute to limited awareness (55) regarding evidence-based clozapine use (51). At the patient level, cultural stigma, low health literacy, and traditional beliefs about mental illness may also act as barriers to clozapine acceptance and adherence (51, 56). These findings align with reports from both high- and middle-income countries, which highlight that systemic and educational challenges remain key contributors to clozapine underutilization worldwide (57, 58).

The widespread use of APP in the MENAT and EMRO regions has critical clinical implications. Polypharmacy is associated with increased risk of metabolic syndrome, drug-induced movement disorders, drug–drug interactions, and higher treatment costs, all of which may contribute to poorer adherence and worse long-term outcomes (14–16). Substituting evidence-based clozapine therapy with multiple antipsychotics may further deprive patients of the most effective treatment for TRS. The high prevalence of APP in MENAT contrasts with international guideline recommendations. This suggests that current treatment guidelines and pharmacological strategies may not fully address the realities of clinical practice. To address these issues, regional health authorities and professional bodies should develop evidence-based prescribing guidelines tailored to local healthcare systems. Coordinated policy measures are essential, including facilitating clozapine prescribing and monitoring, implementing clinician training programs, developing culturally adapted treatment protocols, public education initiatives to reduce stigma, and improving access through subsidized medication plans. Strengthening psychiatric training, promoting the prescribing and monitoring of clozapine, and implementing audit-and-feedback mechanisms may help align regional practices with global standards. By addressing these systematic and educational barriers, clinicians may be less inclined to substitute evidence-based clozapine treatment with polypharmacy regimens of uncertain efficacy and safety (51).

This review offers the first quantitative synthesis of APP prevalence across MENAT and EMRO countries, offering essential insights into regional prescribing practices. The inclusion of studies from multiple countries and diverse clinical settings strengthens the representativeness of the findings. Several limitations should nevertheless be acknowledged. Most included studies were cross-sectional, limiting causal inference regarding factors associated with APP. Heterogeneity in diagnostic criteria, treatment duration, and definitions of polypharmacy may have contributed to the substantial statistical heterogeneity observed. In addition, the small number of studies and the reliance on single studies from several countries limit the strength of the conclusions. Finally, estimates of clozapine utilization may not reflect actual use in the region, as the prevalence was a secondary outcome derived from studies primarily designed to assess APP rather than clozapine prescribing specifically.

## 5 Conclusions

This systematic review and meta-analysis synthesized the available evidence on APP in patients with schizophrenia and other psychotic disorders in MENAT and EMRO regions. The pooled prevalence of APP indicated that approximately one in two patients is concurrently treated with multiple antipsychotics, which is higher than global estimates. Within the included studies on APP, only 12% of patients with schizophrenia and other psychotic disorders were treated with clozapine, which is suggestive of underutilization and possibly substitution of evidence-based clozapine treatment with APP in patients with TRS. Our findings highlight a significant gap between clinical practice and the recommended use of antipsychotic monotherapy. We found limited evidence that specific clinical and treatment-related factors were associated with APP in the systematic review. These factors included higher numbers of hospitalizations, increased relapses, longer disease duration, and the use of depot antipsychotics and anticholinergic medications.

Given the potential risks of APP, such as increased adverse effects and higher costs, urgent action is needed to align regional practices with global recommendations. Policymakers should focus on establishing national prescribing guidelines, strengthening clozapine prescribing and monitoring, and integrating evidence-based pharmacotherapy training into psychiatric education. Future research in the region should focus on prospective, multicenter studies evaluating the clinical outcomes, indications, safety, and cost-effectiveness of pharmacological interventions in patients with schizophrenia.
