# Supplementary material for: Prevalence of antipsychotic polypharmacy in patients with schizophrenia and other psychotic disorders in the MENAT and EMRO countries: a systematic review and meta-analysis
Source: Front Psychiatry. 2026 Apr 29;17:1792876. doi: 10.3389/fpsyt.2026.1792876 (PMC13167998; doi:10.3389/fpsyt.2026.1792876)
Supplement: Supplementary file 1 [file SupplementaryFile1.docx]

**SUPPLIMENTARY MATERIALS**

**Prevalence of Antipsychotic Polypharmacy in Patients with Schizophrenia and Other Psychotic Disorders in the MENAT and EMRO Countries: A Systematic Review and Meta-Analysis**

**Mohammed A. Alhassan^1^*^†^, Mohammed A. Alarabi^2†^, Waled M. Albalawi^3^, Thenuwara Arachchige Omila Kasun Meetiyagoda^4^, Anum Nisar^5^, Wondim Ayenew^6^, Fatimah A Shehab^7^, Gary Remington^8,9,10#^**

^1^ Department of Medical Specialties, College of Medicine, Majmaah University, Al Majmaah, 11952, Saudi Arabia.

^2^ Department of Psychiatry, College of Medicine, King Saud University, Riyadh, Saudi Arabia.

^3^ King Salman Armed Forces Hospital, Tabuk, Saudi Arabia.

^4^ Saitama University, 255 Shimo-okubo, Sakura-ku, Saitama, 338-8570, Japan

^5^ Institute of Population Health, University of Liverpool, Liverpool, UK

^6^ Department of Social and Administrative Pharmacy, School of Pharmacy, College of Medicine and Health Sciences, University of Gondar, Gondar, Ethiopia.

^7^ King Saud Medical City, Riyadh, Saudi Arabia

^8^ Schizophrenia Division, Centre for Addiction and Mental Health (CAMH), Toronto, Canada

^9^ Department of Psychiatry, University of Toronto, Toronto, Canada

^10^ Institute of Medical Sciences, University of Toronto, Toronto, Canada

AUTHOR NOTES:
† These authors share first authorship
# Senior Author

*** Correspondence:**Mohammed A. Alhassan (ORCIR - 0000-0002-3009-7187)
malhassan@mu.edu.sa

**Table S1:** Detailed search strategy, restrictions, access dates, and results for each database.

| **Database** | **Restrictions** | **Access date** | **Search strategy** | **No. of results** |
| --- | --- | --- | --- | --- |
| **OVID Embase** | Human | February 2026 | ((antipsychotic* or neuroleptic* or clozapine* or polypharmacy or combination therapy or multiple antipsychotics or adjunctive or comedication or coprescription*).ti,ab. or exp Antipsychotic Agents/ or exp Neuroleptic Agents/ or exp Clozapine/ or exp Polypharmacy/) and ((schizophrenia or schizophreniform or psychosis or psychotic disorder* or schizophrenic disorder* or schizoaffective or psychotic illness).ti,ab. or exp Schizophrenia/ or exp Schizophreniform Disorders/ or exp Psychotic Disorders/) and (MENAT or EMRO or Middle East* or North Africa* or Eastern Mediterranean or Afghanistan* or Bahrain or Cyprus or Djibouti or Egypt* or Iran* or Iraq* or Israel or Jordan* or Kuwait or Lebanon* or Libya* or Morocco or Oman* or Pakistan* or Palestine or Qatar* or Saudi Arabia* or Somalia* or Sudan* or Syria* or Tunisia* or Turkey or Turkiye or United Arab Emirates or UAE or Yemen or Algeria* or Armenia* or Azerbaijan* or Mauritania*).ti,ab. | 883 |
| **OVID MEDLINE** | Human | February 2026 | ((antipsychotic* or neuroleptic* or clozapine* or polypharmacy or combination therapy or multiple antipsychotics or adjunctive or comedication or coprescription*).ti,ab. or exp Antipsychotic Agents/ or exp Neuroleptic Agents/ or exp Clozapine/ or exp Polypharmacy/) and ((schizophrenia or schizophreniform or psychosis or psychotic disorder* or schizophrenic disorder* or schizoaffective or psychotic illness).ti,ab. or exp Schizophrenia/ or exp Schizophreniform Disorders/ or exp Psychotic Disorders/) and (MENAT or EMRO or Middle East* or North Africa* or Eastern Mediterranean or Afghanistan* or Bahrain or Cyprus or Djibouti or Egypt* or Iran* or Iraq* or Israel or Jordan* or Kuwait or Lebanon* or Libya* or Morocco or Oman* or Pakistan* or Palestine or Qatar* or Saudi Arabia* or Somalia* or Sudan* or Syria* or Tunisia* or Turkey or Turkiye or United Arab Emirates or UAE or Yemen or Algeria* or Armenia* or Azerbaijan* or Mauritania*).ti,ab. | 222 |
| **OVID PsycINFO** | Human | February 2026 | ((antipsychotic* or neuroleptic* or clozapine* or polypharmacy or combination therapy or multiple antipsychotics or adjunctive or comedication or coprescription*).ti,ab. or exp Antipsychotic Agents/ or exp Neuroleptic Agents/ or exp Clozapine/ or exp Polypharmacy/) and ((schizophrenia or schizophreniform or psychosis or psychotic disorder* or schizophrenic disorder* or schizoaffective or psychotic illness).ti,ab. or exp Schizophrenia/ or exp Schizophreniform Disorders/ or exp Psychotic Disorders/) and (MENAT or EMRO or Middle East* or North Africa* or Eastern Mediterranean or Afghanistan* or Bahrain or Cyprus or Djibouti or Egypt* or Iran* or Iraq* or Israel or Jordan* or Kuwait or Lebanon* or Libya* or Morocco or Oman* or Pakistan* or Palestine or Qatar* or Saudi Arabia* or Somalia* or Sudan* or Syria* or Tunisia* or Turkey or Turkiye or United Arab Emirates or UAE or Yemen or Algeria* or Armenia* or Azerbaijan* or Mauritania*).ti,ab. | 186 |
| **Scopus** | Human | February 2026 | (TITLE-ABS-KEY(antipsychotic* OR neuroleptic* OR clozapine* OR polypharmacy OR "combination therapy" OR "multiple antipsychotics" OR adjunctive OR comedication OR coprescription*))  AND  (TITLE-ABS-KEY(schizophrenia OR schizophreniform OR psychosis OR "psychotic disorder*" OR "schizophrenic disorder*" OR schizoaffective OR "psychotic illness"))  AND  (TITLE-ABS-KEY(MENAT OR EMRO OR "Middle East*" OR "North Africa*" OR "Eastern Mediterranean" OR Afghanistan* OR Bahrain* OR Cyprus OR Djibouti OR Egypt* OR Iran* OR Iraq OR Israel OR Jordan* OR Kuwait OR Lebanon OR Libya OR Morocco OR Oman* OR Pakistan* OR Palestine OR Qatar* OR "Saudi Arabia*" OR Somalia OR Sudan* OR Syria* OR Tunisia* OR Turkey OR Türkiye OR "United Arab Emirates" OR UAE OR Yemen OR Algeria OR Armenia OR Azerbaijan OR Mauritania)) | 706 |
| **Web of Science** |  | February 2026 | TS=((antipsychotic* OR neuroleptic* OR clozapine* OR polypharmacy OR "combination therapy" OR "multiple antipsychotics" OR adjunctive OR comedication OR coprescription*)  AND  (schizophrenia OR schizophreniform OR psychosis OR "psychotic disorder*" OR "schizophrenic disorder*" OR schizoaffective OR "psychotic illness")  AND  (MENAT OR EMRO OR "Middle East*" OR "North Africa*" OR "Eastern Mediterranean" OR Afghanistan* OR Bahrain OR Cyprus OR Djibouti OR Egypt* OR Iran* OR Iraq* OR Israel OR Jordan* OR Kuwait OR Lebanon* OR Libya* OR Morocco OR Oman* OR Pakistan* OR Palestine OR Qatar* OR "Saudi Arabia*" OR Somalia* OR Sudan* OR Syria* OR Tunisia* OR Turkey OR Turkiye OR "United Arab Emirates" OR UAE OR Yemen OR Algeria* OR Armenia* OR Azerbaijan* OR Mauritania*)) | 373 |
| **ClinicalTrials.gov** | Human | February 2026 | (antipsychotic* OR neuroleptic* OR clozapine* OR polypharmacy OR "combination therapy" OR "multiple antipsychotic*" OR adjunctive OR comedication OR coprescription*)  AND  (schizophrenia OR schizophreniform OR psychosis OR "psychotic disorder*" OR "schizophrenic disorder*" OR schizoaffective OR "psychotic illness")  AND  (MENAT OR EMRO OR "Middle East*" OR "North Africa*" OR "Eastern Mediterranean" OR Afghanistan OR Bahrain OR Cyprus OR Djibouti OR Egypt OR Iran OR Iraq OR Israel OR Jordan OR Kuwait OR Lebanon OR Libya OR Morocco OR Oman OR Pakistan OR Palestine OR Qatar OR "Saudi Arabia" OR Somalia OR Sudan OR Syria OR Tunisia OR Turkey OR Turkiye OR "United Arab Emirates" OR UAE OR Yemen OR Algeria OR Armenia OR Azerbaijan OR Mauritania) | 66 |
| **PubMed** | Human | February 2026 | (  (antipsychotic*[tiab] OR neuroleptic*[tiab] OR clozapine*[tiab] OR polypharmacy[tiab] OR "combination therapy"[tiab] OR "multiple antipsychotics"[tiab] OR adjunctive[tiab] OR comedication[tiab] OR coprescription*[tiab])  OR  ("Antipsychotic Agents"[Mesh] OR "Neuroleptic Agents"[Mesh] OR "Clozapine"[Mesh] OR "Polypharmacy"[Mesh])  )  AND  (  (schizophrenia[tiab] OR schizophreniform[tiab] OR psychosis[tiab] OR "psychotic disorder*"[tiab] OR "schizophrenic disorder*"[tiab] OR schizoaffective[tiab] OR "psychotic illness"[tiab])  OR  ("Schizophrenia"[Mesh] OR "Schizophreniform Disorders"[Mesh] OR "Psychotic Disorders"[Mesh])  )  AND  (  MENAT[tiab] OR EMRO[tiab] OR "Middle East*"[tiab] OR "North Africa*"[tiab] OR "Eastern Mediterranean"[tiab] OR Afghanistan*[tiab] OR Bahrain[tiab] OR Cyprus[tiab] OR Djibouti[tiab] OR Egypt*[tiab] OR Iran*[tiab] OR Iraq*[tiab] OR Israel[tiab] OR Jordan*[tiab] OR Kuwait[tiab] OR Lebanon*[tiab] OR Libya*[tiab] OR Morocco[tiab] OR Oman*[tiab] OR Pakistan*[tiab] OR Palestine[tiab] OR Qatar*[tiab] OR "Saudi Arabia*"[tiab] OR Somalia*[tiab] OR Sudan*[tiab] OR Syria*[tiab] OR Tunisia*[tiab] OR Turkey[tiab] OR Turkiye[tiab] OR "United Arab Emirates"[tiab] OR UAE[tiab] OR Yemen[tiab] OR Algeria*[tiab] OR Armenia*[tiab] OR Azerbaijan*[tiab] OR Mauritania*[tiab]  ) | 218 |
| **QScience** |  | February 2026 | (All Fields including Full Text contains ‘( antipsychotic* OR neuroleptic* OR clozapine* OR polypharmacy OR "combination therapy" OR "multiple antipsychotic*" OR adjunctive OR comedication OR coprescription* OR "Antipsychotic Agents" OR "Neuroleptic Agents" OR Clozapine OR Polypharmacy ) AND ( schizophrenia OR schizophreniform OR psychosis OR "psychotic disorder*" OR "schizophrenic disorder*" OR schizoaffective OR "psychotic illness*" OR "Schizophrenia" OR "Schizophreniform Disorders" OR "Psychotic Disorders" ) AND ( MENAT OR EMRO OR "Middle East*" OR "North Africa*" OR "Eastern Mediterranean" OR Afghanistan* OR Bahrain* OR Cyprus OR Djibouti OR Egypt* OR Iran* OR Iraq OR Israel OR Jordan* OR Kuwait OR Lebanon OR Libya OR Morocco OR Oman* OR Pakistan* OR Palestine OR Qatar* OR "Saudi Arabia*" OR Somalia OR Sudan* OR Syria* OR Tunisia* OR Turkey OR Türkiye OR "United Arab Emirates" OR UAE OR Yemen OR Algeria OR Armenia OR Azerbaijan OR Mauritania )’) | 19 |
| **Google scholar** |  | February 2026 | (antipsychotic OR neuroleptic OR clozapine OR polypharmacy OR "combination therapy" OR "multiple antipsychotics" OR adjunctive OR comedication OR coprescription OR "antipsychotic agents" OR "neuroleptic agents")  AND  (schizophrenia OR schizophreniform OR psychosis OR "psychotic disorder" OR "schizophrenic disorder" OR schizoaffective OR "psychotic illness")  AND  (MENAT OR EMRO OR "Middle East" OR "North Africa" OR "Eastern Mediterranean" OR Afghanistan OR Bahrain OR Cyprus OR Djibouti OR Egypt OR Iran OR Iraq OR Israel OR Jordan OR Kuwait OR Lebanon OR Libya OR Morocco OR Oman OR Pakistan OR Palestine OR Qatar OR "Saudi Arabia" OR Somalia OR Sudan OR Syria OR Tunisia OR Turkey OR Türkiye OR "United Arab Emirates" OR UAE OR Yemen OR Algeria OR Armenia OR Azerbaijan OR Mauritania) | 61 |
| **Total** | | | | 2734 |

**Table S2:** Patterns of Antipsychotic Use in Studies Reporting Prevalence of Antipsychotic Polypharmacy in Individuals with Schizophrenia and Other Psychotic Disorders in the MENAT and EMRO Regions

| **Author (Year)** | **Country** | **Prescriptions** | **Most Common AP combination used** |
| --- | --- | --- | --- |
| AlDosari et al. (2025) [1] | Saudi Arabia | SGA Monotherapy | Paliperidone, Risperidone, Aripiprazole, Quetiapine |
|  |  | SGA + FGA | Aripiprazole + Zuclopenthixol  Quetiapine + Haloperidol  Olanzapine + Chlorpromazine |
|  |  | SGA + SGA | Aripiprazole + Risperidone  Quetiapine + Aripiprazole  Paliperidone + Olanzapine  Aripiprazole + Paliperidone  Aripiprazole + Olanzapine |
|  |  | ≥ 3 AP | Paliperidone + Olanzapine + Chlorpromazine  Olanzapine + Aripiprazole + Quetiapine  Risperidone + Aripiprazole + Paliperidone  Risperidone + Aripiprazole + Olanzapine |
| Alkhadhari et al. (2015) [2] | Egypt | SGA Monotherapy | Risperidone, Olanzapine, Quetiapine |
|  |  | SGA + FGA | Combinations NR |
|  |  | SGA + Non-AP | Combinations NR |
|  |  | SGA + FGA + Non-AP | Combinations NR |
| Alkhadhari et al. (2015) [2] | Saudi Arabia | SGA Monotherapy | Olanzapine, Risperidone, Quetiapine |
|  |  | SGA + FGA | Combinations NR |
|  |  | SGA + Non-AP | Combinations NR |
|  |  | SGA + FGA + Non-AP | Combinations NR |
| Atik et al. (2008) [3] | Turkey | FGA | Patients with ≥1 FGA |
|  |  | SGA | Patients with ≥1 SGA |
|  |  | SGA + FGA | Combinations NR |
| Dagdemir et al. (2025) [4] | Turkey | FGA + FGA | Combinations NR |
|  |  | SGA + FGA | Combinations NR |
|  |  | SGA + SGA | Combinations NR |
| Kahve et al. (2020) [5] | Turkey | SGA + FGA | Combinations NR |
|  |  | SGA + SGA | Combinations NR |
| Khan et al. (2018) [6] | Pakistan | FGA Monotheraphy | Haloperidol, Prochlorperazine |
|  |  | SGA Monotheraphy | Risperidone, Olanzapine |
|  |  | FGA + FGA | Haloperidol + Fluphenazine |
|  |  | SGA + FGA | Risperidone + Fluphenazine |
|  |  | SGA + SGA | Risperidone + Olanzapine |
| Khdour et al. (2022) [7] | Palestine | FGA | Patients with ≥1 FGA |
|  |  | SGA | Patients with ≥1 SGA |
| Sweileh et al. (2013) [8] | Palestine | FGA Monotheraphy | Combinations NR |
|  |  | SGA Monotheraphy | Combinations NR |
|  |  | FGA + FGA | Combinations NR |
|  |  | SGA + FGA | Combinations NR |
|  |  | SGA + SGA | Combinations NR |
|  |  | ≥ 3 AP | Combinations NR |

**AP** – Antipsychotics, **FGA** - First-generation antipsychotics, **SGA** - Second-generation antipsychotics, **NR** – Not reported

**References**

1. AlDosari F, AlGhossen S, Alburaiki M, Ar Shilbayeh S, AlBogami N, Binsaleh AY, et al. Description of schizophrenia treatment outcomes in Saudi Arabia: A preliminary pilot investigation. J Int Med Res. 2025;53:03000605251332443. https://doi.org/10.1177/03000605251332443

2. Simpson C, Alkhadhari S, Al Zain N, Darwish Selim T, Khan S, Okasha T, et al. Use of second-generation antipsychotics in&nbsp;the&nbsp;acute inpatient management of schizophrenia in the Middle East. NDT. 2015;915. https://doi.org/10.2147/NDT.S78788

3. Atik L, Erdogan A, Karaahmet E, Saraclı O, Atasoy N, Kurcer MA, et al. Antipsychotic prescriptions in a university hospital outpatient population in Turkey: A retrospective database analysis, 2005–2006. Progress in Neuro-Psychopharmacology and Biological Psychiatry. 2008;32:968–74. https://doi.org/10.1016/j.pnpbp.2007.12.031

4. Dağdemir E, Ceyhun HA, Okutucu FT. Antipsychotic treatment patterns and predictors of polypharmacy in psychotic disorders: 20-year real-world data from a tertiary center. Ir J Med Sci [Internet]. 2025 [cited 2026 Mar 5]; https://doi.org/10.1007/s11845-025-04228-4

5. Civan Kahve A, Kaya H, Gül Çakıl A, Ünverdi Bıçakçı E, Göksel P, Göka E, et al. Multiple antipsychotics use in patients with schizophrenia: Why do we use it, what are the results from patient follow-ups? Asian Journal of Psychiatry. 2020;52:102063. https://doi.org/10.1016/j.ajp.2020.102063

6. Khan A, Iqbal Z, Sultan SM, Nazar Z, Tariq M. Antipsychotic prescription patterns and treatment costs of schizophrenia in northwestern Pakistan: A one-year observational study. Trop J Pharm Res. 2018;17:339. https://doi.org/10.4314/tjpr.v17i2.20

7. Khdour M, Salman A. Evaluation of antipsychotic medication adherence and its relation to negative and positive psychiatric symptoms. Journal of Pharmaceutical Health Services Research. 2022;13:224–9. https://doi.org/10.1093/jphsr/rmac019

8. Sweileh WM, Odeh JB, Zyoud SH, Sawalha AF, Ihbeasheh MS. Conformance to schizophrenia treatment guidelines in North West-Bank, Palestine: focus on antipsychotic dosing and polytherapy. BMC Psychiatry. 2013;13:179. https://doi.org/10.1186/1471-244X-13-179
